# A modified multiple-criteria decision-making approach based on a protein-protein interaction network to diagnose latent tuberculosis

**DOI:** 10.1186/s12911-024-02668-z

**Published:** 2024-10-30

**Authors:** Somayeh Ayalvari, Marjan Kaedi, Mohammadreza Sehhati

**Affiliations:** 1 Faculty of Computer Engineering, University of Isfahan, Isfahan, Iran; 2 Department of Biomedical Engineering, School of Advanced Medical Technology, Isfahan University of Medical Sciences, Isfahan, Iran

**Keywords:** Latent tuberculosis infection diagnosis, Multiple-criteria decision-making, Protein-protein interaction, Data fusion

## Abstract

**Background:**

DNA microarrays provide informative data for transcriptional profiling and identifying gene expression signatures to help prevent progression of latent tuberculosis infection (LTBI) to active disease. However, constructing a prognostic model for distinguishing LTBI from active tuberculosis (ATB) is very challenging due to the noisy nature of data and lack of a generally stable analysis approach.

**Methods:**

In the present study, we proposed an accurate predictive model with the help of data fusion at the decision level. In this regard, results of filter feature selection and wrapper feature selection techniques were combined with multiple-criteria decision-making (MCDM) methods to select 10 genes from six microarray datasets that can be the most discriminative genes for diagnosing tuberculosis cases. As the main contribution of this study, the final ranking function was constructed by combining protein-protein interaction (PPI) network with an MCDM method (called Decision-making Trial and Evaluation Laboratory or DEMATEL) to improve the feature ranking approach.

**Results:**

By applying data fusion at the decision level on the 10 introduced genes in terms of fusion of classifiers of random forests (RF) and k-nearest neighbors (KNN) regarding Yager’s theory, the proposed algorithm reached a sensitivity of 0.97, specificity of 0.90, and accuracy of 0.95. Finally, with the help of cumulative clustering, the genes involved in the diagnosis of latent and activated tuberculosis have been introduced.

**Conclusions:**

The combination of MCDM methods and PPI networks can significantly improve the diagnosis different states of tuberculosis.

**Clinical trial number:**

Not applicable.

**Supplementary Information:**

The online version contains supplementary material available at 10.1186/s12911-024-02668-z.

## Introduction

Tuberculosis is a common infectious disease, with a high mortality rate that commonly caused by Mycobacterium tuberculosis, a species of mycobacteria. Conventional methods for diagnosing active tuberculosis (ATB) include skin tests, blood tests, sputum tests, sputum cultures, and chest radiographs [1]. However, these techniques may fail because tuberculosis infections are latent and asymptomatic most of the time. Genetic factors in a person with latent tuberculosis bacteria may play an essential role in developing ATB. It is an attractive research area in studying the human immune system. Over time, as the person’s immune system weakens, the bacteria may wake up, and the person may develop ATB [1]. If ATB stays in the body for a while or is not adequately treated, it may turn into drug-resistant tuberculosis, a condition where the body does not respond to medication. Thus, early prognosis of latent and ATB is beneficial for improving clinical research plans [1–3].

Gene expression that is the process by which information within a gene is used to obtain a functional product, can be measured by different technologies like microarray data and RNA sequencing [4]. In gene expression, data from the activity of thousands of genes are measured and assessed to form an image of cell function. This process determines how cells respond to a disease or treatment [5]. The analysis of gene expression data in Sun et al.‘s study showed that the genes responsible for the activation or hiding of tuberculosis tend to be enriched in different clusters. The genes introduced in this research have the ability to identify tuberculosis disease in different stages [6]. In the study of Deng et al., the gene expression data of 123 patients with tuberculosis were analyzed and 24 genes were identified as the cause of tuberculosis activation [7]. Bah et al.‘s study showed that the genes responsible for tuberculosis disease in adults and children have significant differences [8].

In the study of Tavasoli et al.‘s, a new weighting method is presented to select the best subset of genes. This approach has used the combination of five feature selection methods and different gene ranking methods [9].

The primary purpose of this study is to provide a method for selecting the appropriate feature and classifier for the diagnosis of latent tuberculosis with high reliability. Machine learning algorithms are valuable tools for classifying transcription data. In Multiple Criteria Decision Making (MCDM), the best criteria are selected from among several criteria [10].

The aim of this study is mainly to provide a highly reliable method for diagnosing latent tuberculosis by selecting appropriate feature selection methods and classifiers. For this purpose, we try to identify which feature selection criteria (MIM [11], FDR [12], MIFS [11], correlation coefficient, ANOVA [13–15], entropy, CMIM [11], Ave, Gen-score [16], and JMI [11]) and fusing the results of which classifiers can improve the accuracy of identifying latent tuberculosis-distinguishing genes from healthy control and ATB. MCDM models Linear Assignment, Decision-making Trial and Evaluation Laboratory (DEMATEL)) are applied, and the appropriate feature selection criteria and best classifiers are selected. Filter feature selection methods (t-test, fisher’s discriminant ratio (FDR) [12], analysis of variance (ANOVA) [13–15], Mutual Information Maximization (MIM) [11], mutual Information feature selection (MIFS) [11], joint mutual information (JMI) [11], conditional mutual info maximization (CMIM) [11], entropy, correlation coefficient, and Ave) were used to rank all genes. Using Sequential Forward Feature Selection (SFFS) [17, 18] (as a wrapper feature selection method), optimal combinations of genes were introduced that are the most distinctive regarding Random Forests (RF) classifier. This ranking method was combined with DEMATEL and MCDM method to select limited genes. Finally, 10 genes are introduced that can differentiate between types of tuberculosis.

The main weakness of previous studies is the low accuracy of previous biomarkers, not discriminative for latent tuberculosis infection (LTBI), non-stable due to small overlap of genes among studies and the lack of integration of helpful information such as the Protein-protein interaction (PPI) network before making a decision that motivates our study.

This study utilizes PPI scores to construct a ranking matrix in DEMATEL analysis. DEMATEL is an analytical method used to assess mutual impacts among factors in decision-making processes. PPI refers to intracellular protein collaborations crucial for biological processes. Utilizing DEMATEL alongside PPI enhances the quality of biological analysis, despite potential time complexity, leading to error reduction and improved predictions.

Section 2 is dedicated to the related work. Section 3 describes the feature selection used in this study and introduces data fusion method, and multiple-attribute decision-making method. Section 4 presents the dataset. In Sect. 5, the proposed method is presented. The results are discussed in Sect. 6. Finally, Sect. 7 concludes the paper with a summary.

## Related work

In this section, first, the previous studies related to tuberculosis diagnosis by analyzing the gene expression data are reviewed. Then, the studies related to the diagnosis of other diseases using data fusion are discussed. Afterward, applying MCDM method and different feature selection methods in diagnosis of various diseases are reviewed.

### Tuberculosis diagnosis by analyzing the gene expression data

In the study of Sun et al. [6], by creating molecular networks and studying the interaction of proteins, several genes have been introduced. It has been shown that the identified genes and gene pairs can diagnose tuberculosis patients at different stages of the disease. In the study of Deng et al. [7], 24 genes were identified that could predict the tuberculosis activation. According to the in-depth biological analyses of these 24 genes, 24 signature genes were found, which were capable of predicting ATB, and the production of cytokine was a crucial procedure in the course of the activation of tuberculosis. It is essential to study the signature genes, such as TSPO, CYBB, STAT1, and CD36, further. In recent years, the expression of human genes in response to active/latent tuberculosis has been investigated by Wang et al. [19] and Bah et al. [8], who concluded that host genes often do not exhibit much expression changes in the latent tuberculosis infection scenario. In the study of Juan Zhang et al. [20], the focus was on identifying potential biomarkers for diagnosing tuberculosis in blood and their role in mycobacterium tuberculosis-infected macrophages. Initially, Weighted Correlation Network Analysis (WGCNA) of 9451 genes revealed significant changes in tuberculosis patients’ whole blood. Subsequently, 220 interferon-gamma-related genes were identified, with 30 key genes prioritized using cytoscape. The Area Under the Curve (AUC) values for these genes were calculated for better feature selection. Nine genes were identified, among which SAMD9L showed high diagnostic value (AUC = 0.925) and significant discriminative ability (AUC > 0.865) in ROC analysis. In the study of Wu et al. [21], the objective was to identify diagnostic biomarkers for tuberculosis. Gene ontology analysis indicated significant changes primarily in cell-cell adhesion regulation and T cell activation. KEGG analysis showed host response in tuberculosis primarily involves cytokine-receptor interactions and folate biosynthesis. Using protein-protein interaction networks, IRF1 was identified as a biomarker. Validation in datasets showed increased IRF1 levels in tuberculosis patients compared to healthy individuals. ELISA confirmed IRF1 as a significant biomarker with AUC = 0.801, suggesting its potential use as a new marker for pulmonary tuberculosis diagnosis. In the study of Natarajan et al. [22], integrated analysis identified transcriptional profiles and gene expression signatures distinguishing ATB from latent tuberculosis infection. Pathway analysis indicated upregulated genes are associated with signaling pathways such as IFN and interleukin-1 production. Furthermore, seven-gene signature was proposed as biomarkers for distinguishing between ATB and LTBI, demonstrating high diagnostic accuracy in ROC analysis. In the study of Liu et al. [23], the investigation focused on ATB, caused by mycobacterium tuberculosis. This study provided utilizes several machine learning methods as backpropagation neural network, WGCNA, Single Sample Gene Set Enrichment Analysis (ssGSEA). The study combines multiple machine learning approaches and gene data analysis techniques to develop and evaluate predictive and diagnostic models. In the study of Dai et al. [24], six genes (CASP1, TNFSF10, CASP4, CASP5, IFI16, and ATF3) with strong diagnostic performance (AUC > 0.7) were identified for distinguishing ATB from LTBI. They focus on developing diagnostic models based on biomarker expression data. RF, Least Absolute Shrinkage and Selection Operator (LASSO), and logistic regression methods were employed to develop these models. Delgobo et al. [25] examined whether mycobacterium tuberculosis directly controls spinal cord commitment. Results indicated mycobacterium tuberculosis can transform human CD34 + cells into monocytes/macrophages, a transformation occurring in vitro without type I or II IFN signaling. Moreover, mycobacterium tuberculosis increased IL-6 response in these cells, and inhibiting IL-6R reduced spinal cord commitment and mycobacterium tuberculosis growth in vitro. Genetic, proteomic, and genomic data analysis showed the IL-6/IL6R/CEBP gene module is associated with disease severity in tuberculosis patients, recently evolved to include neanderthal introgression and human microbe adaptation. Chen et al. [26] used WGCNA to identify central genes used in distinguishing between ATB infection (ATB) and latent tuberculosis disease. Using differential analysis and WGCNA, central genes capable of distinguishing between ATB and LTBI were identified. The results showed that five central genes (FBXO6, ATF3, GBP1, GBP4 and GBP5) were identified as potential markers for the progression of LTBI to ATB, which ROC analysis showed that these genes had high diagnostic accuracy with values ​​under the ROC curve between 0.8 and are 0.9. In the study of Yu et al. [27], the profile of seven genes was presented using the RF model, which can be used as potential markers for distinguishing ATB from LTBI in children (AUC = 0.888). This study, summarizes the development and evaluation of machine learning models such as Support Vector Machine (SVM), RF, and Generalized Linear Models (GLM) using specific gene expression profiles. The aim was to identify cluster-specific genes with high diagnostic potential for tuberculosis. The RF model demonstrated significantly lower residual errors compared to other models. In the study of Chen et al. [28], a prediction model was developed using machine-learning classifiers (RF, GLM, SVM, and XGB), with SVM showing the highest AUC in predicting tuberculosis subtypes among pediatric patients. In the study of Wang et al. [29], blood levels of autophagy-related genes (ARGs) were analyzed to differentiate between ATB and latent tuberculosis infection. Three genes (FOXO1, CCL2, ITGA3) positively correlated with adaptive immune lymphocytes and negatively with myeloid and inflammatory cells. A nomogram using these genes accurately distinguished ATB from LTBI patients in subsequent dataset validations.

### Diagnosis of various diseases using data fusion

In the study of Meng et al. [30], a comprehensive review of data fusion methods based on machine learning is presented. In this study, several requirements have been proposed that are used as criteria to evaluate the performance of existing fusion methods based on machine learning. A new classification based on DST and a convolutional neural network (CNN) is proposed to classify valuable collections. A new neural network classification based on CNN and DS in-depth approach for classifying valuable sets is presented. The study by Olivan et al. [31] provides a comprehensive review of recent developments in data integration and machine learning for industrial forecasting, emphasizing on identifying research trends, appropriate opportunities, and undiscovered challenges. In the study of Ali et al. [32], an intelligent model for predicting heart disease has been proposed using deep learning and data fusion. This model fuse data extracted from sensors and medical records to achieve a reliable diagnosis. In a study by Hu et al. [33], a model based on machine learning for the diagnosis of COVID-19 is presented, which uses a fusion of medical information to diagnose the symptoms of the disease reliably. In the study of Simjanoska et al. [34], a multi-level information fusion approach is proposed to learn a blood pressure predictor model using electrocardiogram sensor data. In a study by Razavifar et al. [35], two methods based on the k-nearest neighbor model have been proposed to find the missing values. The first method uses the local search to find the best value of k, and the second method uses the best k-nearest neighbors (KNN) to find the missing values. The proposed model uses Dempster-Shafer’s Theory (DST) method for the final estimation. In the study of Nachappa et al. [36], the performance of several multi-criteria decision analysis (MCDA) models, machine learning and several fused modeling methods have been investigated. Also, in that study, the Dempster-Shafer method was analyzed. In the study of Razi et al. [37], a decision-level data fusion method is proposed to fuse the results of the classifiers using the DST. Wang et al. [38], designed a distributed intrusion detection system using DST to combine evidence from distributed sensors. In the study of Saeed et al. [39], an automated disease diagnosis system using partial least squares regression for feature selection from a set of deeply extracted features is presented. In the study of Arshad et al. [40], an integrated framework for HGR using the deep neural network and the fuzzy entropy-controlled Skewness approach is presented. In the study of Jee and Namin [41], a model is designed using fuzzy criteria to select the best evidence. This study aims to demonstrate the effect of DST and fuzzy reasoning on improving the accuracy of web spam classification. In the study of Tang et al. [42], a framework based on the fused classification of RF and D-S Evidence Theory for detecting single faults is presented. In the study of Wang et al. [43], a two-step framework, namely, hierarchical fusion hierarchical and heterogeneous fusion, to fuse the results of different classifiers was introduced.

### Diagnosis of various diseases using MCDM

Kim et al. [44] and Hashemi et al. [45] tried to model a feature selection process as a multiple-criteria decision making procedure. Such a technique has employed the TOPSIS (i.e., Technique of Order Preference by Similarity to Ideal Solution) approach for the evaluation of the features on the basis of their association with a number of labels as various criteria. In addition, the Technique of Order Preference by Similarity to the Ideal Solution is meant to include the alternatives featuring the nearest distances to the ideal positive solution while maximizing the distances to the negative one. He et al. [46] introduced an integrated MCDM in order to combine a variety of classifications in the MCDM framework so as to evaluate the comparative weights of various classifiers. Farhadi et al. [47] identified and prioritized the factors contributing to service quality from the views of those healthcare providers employed in the educational hospitals affiliated with the University of Medical Sciences of Shiraz. As an instance, Hsieh et al. [48] presented a case study pertaining to a beverage & food information system.

### Diagnosis of various diseases using different feature selection methods

In the study of Maghsoudloo et al. [12], Feature selection methods such as FDR have been used to diagnose asthma and to introduce biomarkers and genes that cause the disease. In the study of Shrivastava et al. [49], with the help of feature selection method FDR and classifier SVM succeeded in designing a new system for diagnosis and classification of skin diseases psoriasis In the study of Pascal Ezenkwu et al. [50], used a variety of feature selection methods such as SFFS and MI to select features related to the entire dataset. In the study of Xu et al. [51], various feature selection methods have been compared to analyze the decoding of brain states from FMRI data. Two methods of feature selection of Kendall matching coefficient and ANOVA have been used. Yadegaridehkordi et al. [52], used the DEMATEL method in order to discover the interdependencies between the factors as well as their significance in big data. In study of Saghapour et al. [53], a variety of new feature ranking methods have been proposed to predict cancer conditions from protein data. This method includes ten feature selection techniques that were combined with the Topsis method to identify the most distinct proteins to detect different types of cancer.

## Materials and methods

### Feature selection, data fusion, and MCDM

In the proposed method, the concepts of Feature selection, data fusion, and MCDM will be used. For this reason, in the rest of this section, these concepts will be introduced.

#### Feature selection

Feature selection methods are divided into three categories: filter-based methods, wrapper techniques, and hybrid approaches. Filter-based methods are independent of learning algorithms and use statistical data features to select features so that one credit is calculated for each feature. Features are sorted by their rankings, and features with the best ranks are removed. Filter-based methods are fast because they do not use learning algorithms and are suitable for high-dimensional data. Another method of feature selection is the wrapper technique. Subset evaluation provides a subset of candidate features based on a specific search strategy. This technique exploits a machine learning algorithm for effective feature selection. Therefore, this method has high accuracy, but because of using a machine learning algorithm, its computational complexity is also high. Hybrid methods consist of two steps. In the first step, filter-based methods are used to reduce data dimensions, and in the second stage, wrapper methods are applied to select the best subset of features [35, 54, 55].

#### Data fusion

Data fusion is a technique that fuses data collected from different sources. The purpose of this method is to create a predictive model of a system based on data obtained from several sources and classifiers. This study employs data fusion across three levels: data level, feature level, and decision level. At the data level, features (genes) from various microarray datasets are integrated. At the feature level, the IDE feature selection method is applied to identify significant genes based on IDE scores. At the decision level, Dempster-Shafer and Yager methods are utilized to optimize classifier combinations. The levels of data fusion and the strengths and weaknesses of each are presented in Table 1. More details about these three data fusion levels are provided in the rest of this Sects. [35, 41, 56–59].

**Table 1:** Strengths and weaknesses of different levels of data fusion method [56, 59]

Level	Strengths/Weaknesses	Description
Data level	Strengths	Increased accuracy: Combining data from multiple sources can enhance analytical precision. Noise reduction: Data fusion can decrease data noise and improve information quality
	Weaknesses	Computational complexity: Integrating diverse data may require complex and time-consuming processing. Data overlap and interference: Overlapping or conflicting data may create integration challenges.
Features level	Strengths	Optimal feature selection: Information fusion aids in identifying important features for analysis. Dimensionality reduction: Eliminating unnecessary features reduces data dimensions and speeds up analysis.
	Weaknesses	Information loss: Incorrect feature selection can lead to the loss of crucial information. Sensitivity to data changes: Variations in data can affect feature selection and degrade analysis quality.
Decision level	Strengths	Reduced decision overlap: Combining different decisions can mitigate overlap and ambiguity in decision-making. Improved accuracy and predictability: Decision fusion enhances decision accuracy and predictive capability.
	Weaknesses	Decision-making complexity: Optimal decision combination can be intricate and require extensive study and testing. Potential errors: Incorrect decision fusion may lead to errors and mistakes in decision-making.

##### Data level

In this approach, no analysis or processing is performed on the data. In this method, the data from different sources are directly fused [35, 56].

##### Feature level

At this level, practical information features are extracted from various sources. Features extracted from different sources are fused to form a group of features that will represent the state of a system [35, 56].

###### Improved distance evaluation (IDE)

IDE is a concept used in data fusion, particularly in the context of multi-sensor data fusion or sensor data integration. IDE is a method used to enhance the accuracy of data fusion by considering the distances between the measurements obtained from different sensors or sources. In data fusion, multiple sensors or sources are used to collect data about a particular phenomenon or object. These sensors might have different characteristics, noise levels, or biases. IDE aims to address the challenges of fusing data from diverse sources by evaluating the distances between data points in the combined feature space. The key idea behind IDE is to assess the consistency and reliability of the measurements from different sources. By considering the distances between measurements, the method can identify outliers, inconsistencies, or unreliable data points. This helps in making more informed decisions during the data fusion process and improves the overall quality and accuracy of the fused data. IDE can be particularly useful in scenarios where the sources have different levels of accuracy, noise, or uncertainty. By incorporating distance evaluation, the fusion process can mitigate the impact of unreliable or inconsistent measurements, leading to more robust and accurate results.

IDE method is one of the feature selection methods used in data fusion at feature level. In this method, the best features should have the following condition: their values are as close to each other as possible for one class, and as far apart as possible for two different classes. In this method, using a kind of averaging of the importance ​​of the features and the distance between their placement centers on the page, a score is assigned to each feature. The higher the score of a feature, the higher the values of that feature for two different classes, and the values of that feature are close together for a class. Then, by setting a threshold, features that score above the threshold are selected as the best features. The main idea of this method is explained below:

Suppose that $$\:\text{c}=\text{1,2},\dots\:,\text{C}$$ is number of classes, $$\:m$$$$\:=\text{1,2},\dots\:,M$$ is the number of samples in each class, and $$\:\text{j}$$$$\:=\text{1,2},\dots\:,\text{J}$$ is the number of features extracted. We consider $$\:{q}_{m}$$, $$\:c$$, $$\:j$$ to be the value of the $$\:j$$ feature of the $$\:m$$ sample of class $$\:c$$. The mean distance between the values of a feature that are extracted from different samples of a class is obtained from Eq. (1) [35, 54]:

1 $$\:{d}_{c,j}=\frac{1}{M\times\:\left(M-1\right)}\times\:{\sum\:}_{l,m}^{M}\left|{q}_{m,c,j}-{q}_{l,c,j}\right|;\text{m}\ne\:1$$

The maximum changes of $\:{d}_{c,j}$ are obtained using the following Eq. (2) [35, 57]:

2 $$\:{V}_{j}^{\left(w\right)}=\left|\frac{{max}\left({d}_{c,j}\right)}{{min}\left({d}_{c,j}\right)}\right|$$

The larger the $\:{V}_{j}^{\left(w\right)}$ index for a feature, the greater the distance between the values of that feature in different classes. So the larger the index, the higher the feature score. To calculate the mean distances of feature between different classes, first the average values of a feature for all samples in a class are obtained as follows [35, 57]:

3 $${u_{c,j}} = {1 \over M}\, \times \,\sum \, _{m = 1}^M{q_{m,c,j}}$$

Now the distance between the mean values of a feature in different classes is calculated as follows [35, 57]

4 $$\:{d}_{j}^{\left(b\right)}=\frac{1}{\text{C}\times\:(C-1)}\:\times\:{\sum\:}_{c,e=1}^{C}{|u}_{c,j}-{u}_{e,j}{|}$$

The higher this index is for one feature, the greater the distance of values of that feature are for different classes, and the more appropriate it will be to differentiate between those classes. So far we have worked on the average distance of the classes from each other, but we have not paid attention to the changes. The more variation (variance), the less reliable our system is. That is, the features are close to each other in one class and far from each other in different classes, but with a lot of changes and uncertainty. Therefore, we define the compensation factor or compensation index. The greater the variation ($\:V$), the larger the denominator, the smaller the reward index ($\:\lambda\:$) and the lower the feature score.

The maximum changes in the $\:{d}_{j}^{\left(b\right)}$ index are calculated as follows [35, 57]:

5 $$\:{V}_{j}^{\left(b\right)}=\:\frac{max\left({|u}_{c,j-}{u}_{e,j}\right|)\:}{min\left(\right|{u}_{c,j-}{u}_{e,j}\left|\right)}$$

The greater the distance between the values of a feature within a class, the lower its score. The greater the distance between the values of a feature between different classes, the higher its score. So the score of each feature is proportional to [35, 57]:

6 $$\:{\alpha\:}_{j}\approx\:\frac{{d}_{j}^{\left(b\right)}\:\:}{{d}_{j}^{\left(w\right)}}$$

Any feature that has less variance in the values of $\:{d}_{j}^{\left(b\right)}$ and $\:{d}_{j}^{\left(w\right)}$ indices will have better quality, so it should get more points. Accordingly, using the variances of these values, the reward index is defined for each feature [35, 57]:

7 $$\:{{\uplambda\:}}_{j}=\:\frac{1}{\:\frac{{V}_{j}^{\left(w\right)}}{max\left({V}_{j}^{\left(w\right)}\right)}+\:\frac{{V}_{j}^{\left(b\right)}}{max\left({V}_{j}^{\left(b\right)}\right)}}$$

Using the reward factor, the score of each feature in raw form is equal to [35, 57]:

8 $$\:{\alpha\:}_{j}={\lambda\:}_{j}\:\times\:\frac{{d}_{j}^{\left(b\right)}\:\:}{{d}_{j}^{\left(w\right)}}\:\:$$

Finally, using the following formula, the score of each feature is normalized so that the best features can be selected by applying the desired threshold [35, 57]:

9 $$\overline {\alpha j} = {{{\alpha _j}} \over {\max \left( {{\alpha _j}} \right)}}$$

##### Decision level

This is the highest level in data fusion. There is the highest accuracy and most minor error in data fusion at this level. The largest volume of computation is at this level [35, 57].

###### DST and Yager’s theory

As a generalization of the Bayesian technique, DST allows a typical uncertainty level and paves the way for explicitly accounting for the undetermined potential cause of the observational data [41]. As a well-known evidence theory, it defines the basic probability assignments (BPAs) function in order to present the evidence combination [58]. In addition, belief uncertainty intervals are employed by DST on the basis of the evidence obtained via a number of observations in order to introduce the assumed belief [57]. As an efficient technique for assessing uncertainty and modeling imprecision, the DST is capable of providing more flexibility for the purpose of specifying uncertainty in probabilistic models and testing the hypotheses. Theoretically, two essential functions are necessary for the purpose of displaying information, known as Bel and plausibility function (PLS). PLS and Bel derive the upper bound for an unknown probability function and the lower bound value for a known probability function, respectively. One can decide the uncertainty of the knowledge with regard to the objective proposition by determining the differentiation between PLS and Bel. DST is used to combine data at the decision level. Three essential functions are used in DST [58, 59]:

- The basic function of probability mass (m).
- Belief functions (Bel).
- Plausibility function (PLS).

The basic function of probability mass, called the mass function, is the most critical part of evidence theory and is known by the symbols m and Basic Probability Assignment (BPA). The PLS and Bel functions are the upper and lower limits of the occurrence of a subject, respectively, which are defined based on the basic function of probability mass [41, 57]. In evidence theory, situations where the conflict between classifiers (evidence) is severe, may lead to an entirely erroneous estimate. Yager introduced an efficient method in which the possibility of conflict between the evidence is adequately considered.

10 $$\:\text{B}\text{e}\text{l}\left(\text{A}\right)={\sum\:}_{B|B\subseteq\:A}^{}\text{m}\left(\text{B}\right)\:\:\:\:\text{P}\text{l}\text{s}\left(\text{A}\right)=\:{\sum\:}_{B|B\cap\:A\ne\:\varphi\:}^{}\text{m}\left(\text{B}\right)$$

11 $${\text{Bel}}\left({\text{A}}\right)\:\le\:P\left(A\right)\le\:PlS\left(A\right)\:{\xrightarrow{if\:PlS\left(A\right)=Bel\left(A\right)}}\:bel\left(A\right)=P\left(A\right)=PlS\left(A\right)$$

**Fig. 1:**
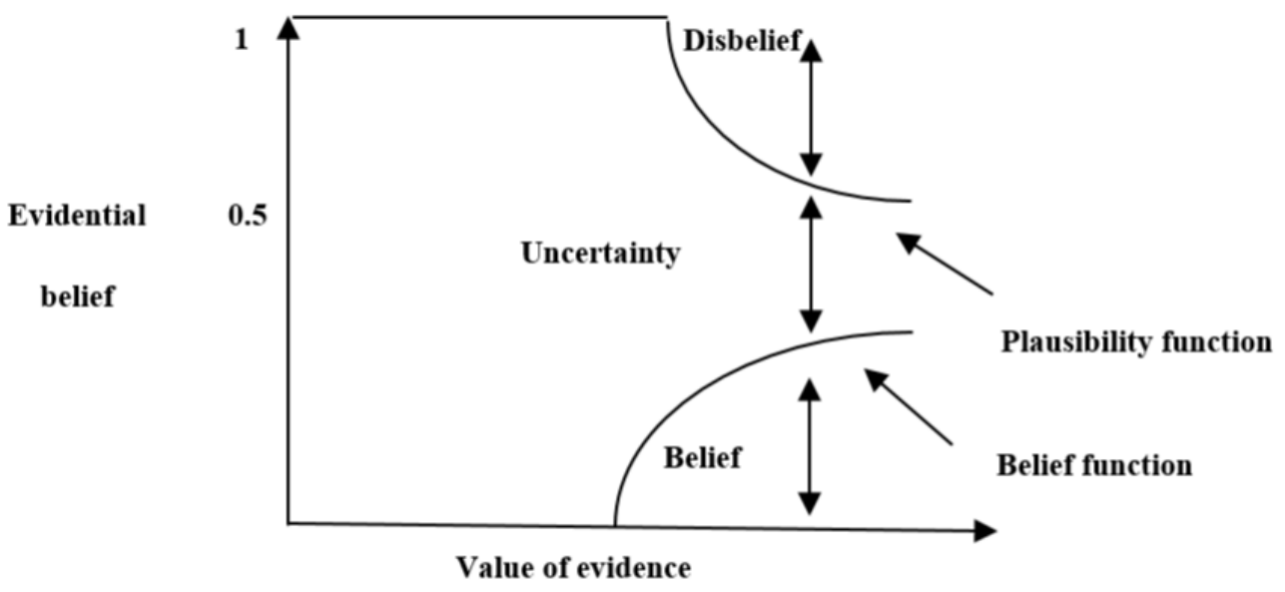
The general DST scheme [59]

The real probability of the occurrence of an event such as $\:A$, denoted by $\:P\left(A\right)$, is a value between the Belief functions and Plausibility function values of that event. Figure 1 shows the schematic of DST combinations.

#### Multiple-criteria decision-making (MCDM)

MCDM is the method of selection when dealing with several different criteria. For example, when we use several classifiers or different feature selection methods to diagnose a disease, after examining and integrating them, we can announce our final diagnosis. Using the principles of MCDM can analyze each criterion separately and create the result. In MCDM, instead of measuring the optimality with one criterion, several different criteria are used so that there is no loss or regret about other criteria. This method provides the possibility of selecting the best solution and making the best decision among multiple alternatives that sometimes even conflict with each other. This decision-making method provides the possibility of selecting between one goal or several goals for a decision maker or several decision makers. It is the process that leads to an answer from among the solutions selected to solve the problem [60–62].

##### Linear assignment

This method is one of the simplest available in multiple-attribute decision-making. Criteria are generally denoted by$\:\:{C}_{j}$, $\:j=\{1,\dots\:,n\}$. Alternatives are usually denoted by $\:{A}_{i}$, $\:i=\{1,\dots\:,m\}$. $\:{X}_{ij}$’s are points or scores that are assigned to the performance of alternatives relative to criteria [60].

If the criterion is incremental, it becomes normal using Eq. (12):

12 $$\:{r}_{ij}=\frac{{x}_{ij}}{{\sum\:}_{\text{i}=1}^{\text{m}}{x}_{ij}}\:\:\:\:\:\:\:or\:\:\:\:{r}_{ij}=\frac{{x}_{ij}}{\text{M}\text{a}\text{x}\:\left\{{x}_{ij}\right\}}$$

If the criterion is decremental, it becomes normal using Eq. (13):

13 $$\:{r}_{ij}=1-{x}_{ij}\:\:\:\:\:or\:\:\:{r}_{ij}=\frac{\text{M}\text{i}\text{n}\:\left\{{x}_{ij}\right\}}{{x}_{ij}}$$

The next step is to form the weighted matrix. In this step, according to the weights calculated from other methods (Shannon entropy and other methods), Linear Assignment obtains the weighted matrix. Finally, the best alternative is selected, and the score of each alternative is calculated by summing the rows of the weight matrix and based on that, alternatives are ranked.

##### DEMATEL

In order to analyze the influence relationships between a system’s factors, The DEMATEL can serve as an effective technique. By analyzing the whole influence relations among the factors via the DEMATEL, one can reach an ideal solution for solving complicated problems of the system and a better perception of the structural relations. DEMATEL is one of the methods in MCDM and a way of structuring the problem based on the opinion of experts or classifiers, which is used here in terms of different classifiers. With the help of a communication table, the vector of superiority and the communication vector of features (genes) are calculated. In this method, the effectiveness and dependence of each feature are obtained. The intensity of the effect of feature $\:i$ on feature $\:j$ is denoted by one of the numbers 4, 3, 2, 1, 0. R indicates the degree of effectiveness and C indicates the dependency degree of each feature.

R + C (Superiority vector): The higher the index value, the more the feature interacts with the other features and the more important that feature is.

R-C (Communication vector): represents the net effect of the feature in the system. If the value of R-C is greater than zero, we have an effective feature, and if it is less than zero, the corresponding feature (gene) is dependent [60–62].

As will be mentioned later, in the current study, the scores of the table presented in Appendix E have been used to create the communication table.

#### Sequential Forward feature selection (SFFS)

SFFS is a feature selection technique used in machine learning and statistics. It’s a method for choosing a subset of relevant features from a larger set of features to improve the performance of a predictive model. In SFFS, the process starts with an empty set of selected features. It iteratively adds one feature at a time, selecting the feature that provides the best improvement in model performance, until a predefined stopping criterion is met. At each step, the algorithm evaluates the performance of the model using cross-validation or some other evaluation metric, and then selects the next feature to add based on its impact on performance. SFFS is a forward selection technique because it starts with no features and incrementally builds up the set of selected features. This method can be effective in reducing the dimensionality of the feature space and improving model accuracy, as it aims to include only the most relevant features while excluding irrelevant or redundant ones. Through the iterative elimination of the worst features or aggregation of the best features, the algorithms of sequential feature selection seek to find an efficient subset of features [17, 18]. By beginning the search from a null/random subset $\:{X}_{0}$, SFFS carries out a process iteratively in order to select the most significant feature (MFS) out of the remaining dataset (at iteration $\:k$: $\:{Y}_{k}$ = $\:U$ - $\:{X}_{k}$), which will be added to $\:{X}_{k}$ ($\:{X}_{k}$ = $\:{X}_{k}$ U $\:MFS$). Subsequently, SFFS continues the repeated process of finding and deleting the least significant features (LFS) from the new subset. Following each phase of iteration, a comparison is made between the obtained results and the results obtained during the preceding step ($\:{X}_{k}$). If we have an improved outcome, then $\:{X}_{k}+1$*=*$\:{X}_{k}$*–*$\:\:LFS$. The same process is iterated until a particular criterion is reached. The MFS and LFS are chosen by applying an evaluation criterion and a wrapper algorithm.

#### PPI

According to recent studies, any functional defect in one of the pathway proteins may indicate biological disorders such as tuberculosis. PPI is a protein interaction identification method and a popular application of scientific visualization techniques. PPIs are extracted from the HIPPIE database [63], which is a very comprehensive repository that integrates information from several well-established databases (such as BioGRID [64] and HPRD [65]). PPI provides an estimated confidence score and is very significant in terms of investigating the functionality of proteins [66–68].

## Dataset

The datasets used in this study are microarray data collected from the national center for biotechnology information (NCBI) [69–72]. Each row in datasets represents a data sample and each column represents a gene. The data samples used in our study are from the three classes of healthy control, ATB, and latent tuberculosis. It should be noted that the original dataset included other classes, but we do not consider them in this study. In the present work, each gene is considered a feature. The details of datasets and the number of their samples are listed in Table A1 in Appendix A. Datasets were reported by their gene expression omnibus series code, gene series expression (GSE).

## The proposed approach

The aim of this study is to identify key genes that distinguish between individuals with LTBI, ATB, and healthy individuals. To achieve this goal, we employ data fusion techniques at both the feature and decision levels, enhancing the reliability of our findings by integrating diverse sources of evidence and information.

Our approach integrates multiple microarray datasets (detailed in Sect. 4) to leverage a combination of Multi-Criteria Decision Making (MCDM) methods and PPI networks for tuberculosis diagnosis. Specifically, we utilize the IDE method for feature-level data fusion and apply Dempster-Shafer and Yager’s theory at the decision level.

**Fig. 2:**
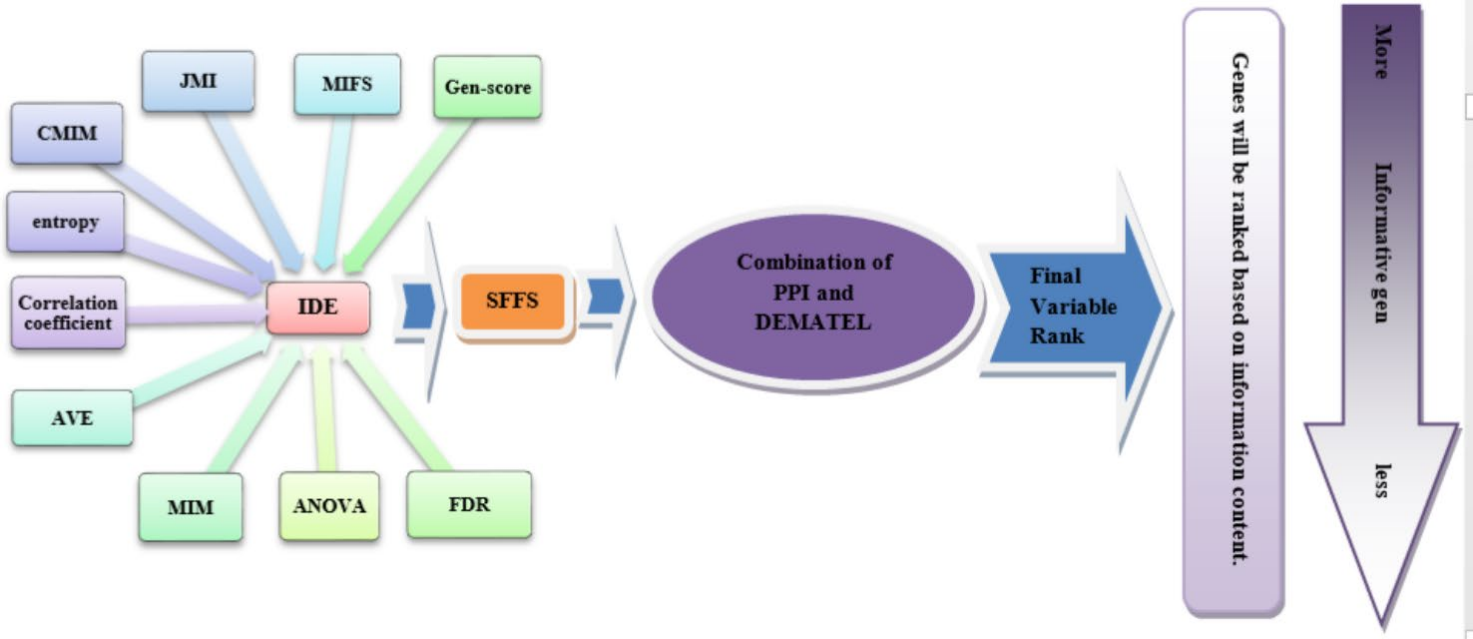
The schematic of the proposed model

**Fig. 3:**
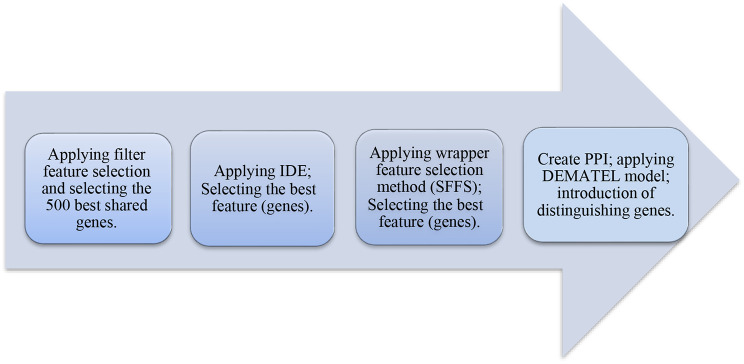
The process of the proposed method

Here is a breakdown of our proposed method (illustrated in Figs. 2 and 3):

1. Feature Selection: We apply filter-based feature selection methods (as shown in Appendix B) to the genes across five microarray datasets. Through 10-fold cross-validation, we identify the top 500 genes consistently selected by all methods from the training datasets. These genes are then tested on dataset GSE 19444 [71].
2. Classifier Evaluation: Using Random Forest (RF), Naïve Bayes (NB), k-Nearest Neighbors (KNN), and Support Vector Machine (SVM), we evaluate the performance of these 500 genes. Results from this step guide the selection of optimal feature selection methods, detailed in Appendix C.
3. Ranking and Selection: The Linear Assignment method is applied to rank classifiers based on the outcomes of Step 2, determining the most effective classifiers.
4. Feature Fusion: The IDE method is used to fuse features selected in Step 1, identifying the most discriminative genes.
5. Optimal Gene Combination: SFFS is employed on genes identified in Step 4 to refine gene selection and determine the optimal combination of genes and classifiers to enhance tuberculosis classification across the microarray datasets.
6. Protein Interaction Analysis: Utilizing the STRING database [73], we construct a PPI network to assess gene interactions. The network is used to generate a communication table for weight matrix construction via the DEMATEL method, ranking the top 236 discriminative genes identified in Step 5.
7. Decision Fusion: Decision-level data fusion is conducted to optimize classifier combinations for classifying microarray data into LTBI, ATB, and healthy states. This fusion approach reveals that integrating RF and KNN classifiers on features selected by criteria like correlation coefficient, MIM [11], MIFS [11], and entropy achieves the highest accuracy. RF and KNN are applied on 26 introduced genes in Step 6.
8. Accumulative clustering and hierarchical clustering: Accumulative clustering and hierarchical clustering are used to introduce pairs of genes responsible for hiding and activating tuberculosis. Common genes between the current study and the study of Sun, et al. [6] and Bah, et al. [8] were examined to introduce the distinguishing genes between the latent and active states of TB on the GSE 19444 [71], and top 10 discriminative genes were identified.
9. Decision Fusion: Finally, RF and KNN are applied on 10 genes introduced in Step 8.

The proposed approach aims to address challenges associated with noisy data in Microarray datasets and the lack of a stable analytical framework.

Feature selection plays a pivotal role by identifying and prioritizing relevant features using various models and algorithms. This process offers several benefits as follows:

### Dimensionality reduction:

Selecting important features reduces data dimensionality, enhancing algorithm efficiency.

### Noise reduction:

Eliminating unnecessary features improves model accuracy and mitigates noise effects in Microarray datasets.

### Enhanced generalization:

Significant features improve algorithm robustness and generalization capabilities.

### Ambiguity reduction:

Selecting appropriate features minimizes data ambiguities, thereby improving distance estimation accuracy.

In data fusion, effective feature selection addresses challenges posed by noisy data through:

### Removing noisy features:

Identifying and removing features associated with data noise improves decision-making and distance evaluation.

### Improving pattern detection:

Focusing on significant features enhances pattern recognition amidst noise.

### Enhancing algorithm robustness:

Eliminating noise in Microarray datasets and irrelevant features improves algorithm performance with new data.

The SFFS method is highlighted for its role in feature selection in machine learning, offering benefits such as noise reduction and improved model performance. However, it should be integrated with other methods for comprehensive noise mitigation and enhanced model accuracy.

Therefore, in the following, PPI and DEMATEL have been used. Additionally, the PPI network approach is recommended for analyzing microarray data due to its ability to study protein networks holistically, detect patterns, predict interactions, and reduce noise in Microarray datasets. This study introduces a novel approach by utilizing PPI degrees to construct a ranking matrix for DEMATEL analysis. DEMATEL is an analytical method for assessing mutual influences among factors in decision-making processes. The PPI refers to collaborations between proteins within cells, crucial for biological processes. Integrating PPI with DEMATEL offers several advantages: identifying and evaluating protein networks, understanding mutual effects between proteins, predicting physiological changes, and managing complexities in biological systems. This combined approach enhances decision-making in biology, medicine, and drug development by providing deeper insights into protein interactions and their implications.

The average degree of connectivity in a protein-protein interaction (PPI) network is defined as the average number of connections (edges) to each node (protein). If $\:\text{V}$ is the number of nodes (proteins) and $\:\text{E}$ is the number of edges (connections), the average degree $\:\langle K\rangle$ is calculated as follows:

14 $$\langle K\rangle \, = \,{{2E} \over V}$$

Here, $\:2\text{E}$ represents the total number of edges in the network because each edge connects two nodes. Dividing $\:2\text{E}$ by $\:\text{V}$ gives the average number of connections per node. This equation indicates the average number of connections each protein has, which is crucial in analyzing complex networks like PPI networks. Here are the simplified equations for using PPI data in constructing the weight matrix for DEMATEL:

**Weight matrix for DEMATEL**

To construct the weight matrix in DEMATEL using PPI data, we define the weights $\:\text{w}\text{i}\text{j}$ as follows:

15 $$\:{{W}_{ij}=PPI}_{ij}$$

Here, $\:{PPI}_{ij}$ represents the positive or negative impact of factor $\:\text{i}$ on factor $\:\text{j}$, derived from protein-protein interactions. If $\:{PPI}_{ij}$ is a positive value, $\:{W}_{ij}$ ​will be a positive value as well, and if $\:{PPI}_{ij}$is a negative value, $\:{W}_{ij}$ will be a negative value too.

**Degree of dependence matrix in DEMATEL**

To calculate the degree of dependence matrix $\:\text{D}\text{E}\text{M}$ in DEMATEL, we use the weight matrix $\:\text{w}$ derived from Eq. (16):

16 $$\:{DEM}_{ij}\:=\frac{{W}_{ij}\:+{W}_{ji}\:}{2}$$

Here, $\:{W}_{ij}$ and $\:\text{w}\text{j}\text{i}$ are weights obtained from PPI data, representing the positive and negative impacts between factors $\:\text{i}$ and $\:\text{j}$.

These equations illustrate how PPI data can be utilized to construct the weight matrix and calculate the degree of dependence matrix in DEMATEL methodology.

This approach results in selecting the top 500 genes from each dataset. Omitting each step in the presented method leads to incorrect results. For example, omitting filter-based feature selection methods and preprocessing before implementing information fusion and IDE feature selection result in a large number of features (genes) entering the IDE method and complicates the analysis of the IDE feature selection method. Omitting the wrapper method in SFFS feature selection prevents us from determining which gene combinations lead to better results. Additionally, omitting feature selection methods and directly using PPI leads to increased noise and overfitting, making the analysis of results difficult. If the PPI scores are not used in DEMATEL, the relationships between genes and their impact on each other may not be properly identified. The ablation study and a more detailed examination of the impact of each step of the proposed method are presented in Sect. 6 (Table 10).

## Experiments

### Evaluation measures

In this study, sensibility, specificity and accuracy have been used to evaluate the proposed method.

A confusion matrix, as an evaluation tool for predictive model performance, particularly in classification problems, is used. In the current study, which aims to identify distinguishing genes of LTBI, this matrix aids in assessing the accuracy of these genes. The matrix consists of four main components:

True Positive (TP): Number of samples correctly classified as LTBI.

True Negative (TN): Number of samples correctly classified as non-LTBI.

False Positive (FP): Number of samples incorrectly classified as LTBI (instead of non-LTBI).

False Negative (FN): Number of samples incorrectly classified as non-LTBI (instead of LTBI).

Based on these four values, various metrics can be calculated to evaluate the accuracy of your model. The most important of these metrics include:

Accuracy: The ratio of correct predictions$\:(TP+TN)$, to the total number of samples$\:(TP+TN+FN+FP)$.

Accuracy is calculated using Eq. 17.

17 $$\bullet\:Accuracy=\frac{TP+TN}{TP+TN+FN+FP}$$

This metric indicates how accurate your model’s classifications are across all classes (latent and non-latent).

Furthermore, from the confusion matrix, other metrics such as Sensitivity and Specificity have also been calculated, which are particularly useful in problems with imbalanced data.

$\:\text{S}\text{e}\text{n}\text{s}\text{i}\text{b}\text{i}\text{l}\text{i}\text{t}\text{y}$ is calculated using Eq. (18).

18 $$\bullet Sensibility=\frac{TP}{TP+FN}$$

Specificity is calculated using Eq. (19).

19 $$\bullet Specificity=\frac{TN}{TN+FN}$$

### Results and discussion

In the rest of this section, the results related to the several steps of the proposed method are presented and discussed.

#### Data fusion at the feature level, and use of the IDE method

Improved Distance Evaluation (IDE) is a method used for feature selection to assess and select important features in various problems such as detecting latent tuberculosis genes. In this approach, the importance of each feature is computed based on the variations and differences present in the data. This approach leads to the selection of features that have a higher capability to differentiate between different data categories, thus enhancing the accuracy and precision of data modeling and analysis. The inputs at this stage include 500 top genes from each dataset, upon which the IDE feature selection method has been applied at the information fusion stage. The best features (genes), as determined by the feature selection method specified in this stage, are identified. The study examines how IDE (Information Density Estimation) can serve as a valuable computational tool for selecting top genes in microarray data analysis. Graphical representation helps researchers make informed decisions about which genes to prioritize for further analysis and investigation. The figures related to IDE are presented in Appendix D. The statistical report of the results, which contains the average and standard deviation (SD) of the importance of features (genes), is shown in Table 2.

**Table 2:** IDE statistical report

	GSE 19439 [70]		GSE 19491 [69]		GSE 39939 [72]		GSE 37250 [72]		GSE 28623 [70]	
	Average	SD	Average	SD	Average	SD	Average	SD	Average	SD
Ave	0.89	0.57	0.94	0.09	0.96	0.09	0.96	0.09	0.75	0.16
Entropy	0.99	0.04	0.95	0.07	0.99	0.04	0.96	0.04	0.96	0.09
Correlation coefficient	0.89	0.57	0.95	0.07	97.5	0.04	0.97	0.03	0.97	0.05
FDR (Fisher’s discriminant ratio) [12]	0.97	0.32	0.97	0.32	0.97	0.32	0.96	0.10	0.92	0.07
ANOVA (analysis of variance) [13–15]	0.97	0.04	0.97	0.04	0.97	0.04	0.97	0.06	0.96	0.0.8
MIM (Mutual Information maximization) [11]	0.95	0.06	0.94	0.09	0.95	0.07	0.97	0.03	0.89	0.13
JMI (Joint Mutual Information) [11]	0.98	0.03	0.95	0.07	0.98	0.06	0.95	0.07	0.97	0.04
CMIM (Conditional Mutual Info Maximization) [11]	0.75	0.17	0.93	0.06	0.95	0.07	0.95	0.06	0.97	0.04
Gene score	0.97	0.04	0.96	0.05	0.95	0.07	0.95	0.07	0.92	0.07
MIFS(Mutual Information feature selection) [11]	0.96	0.09	0.97	0.03	0.97	0.03	0.97	0.07	0.98	0.03

#### Creating ranking matrices using linear assignment and identifying the best classifiers

**Fig. 4:**
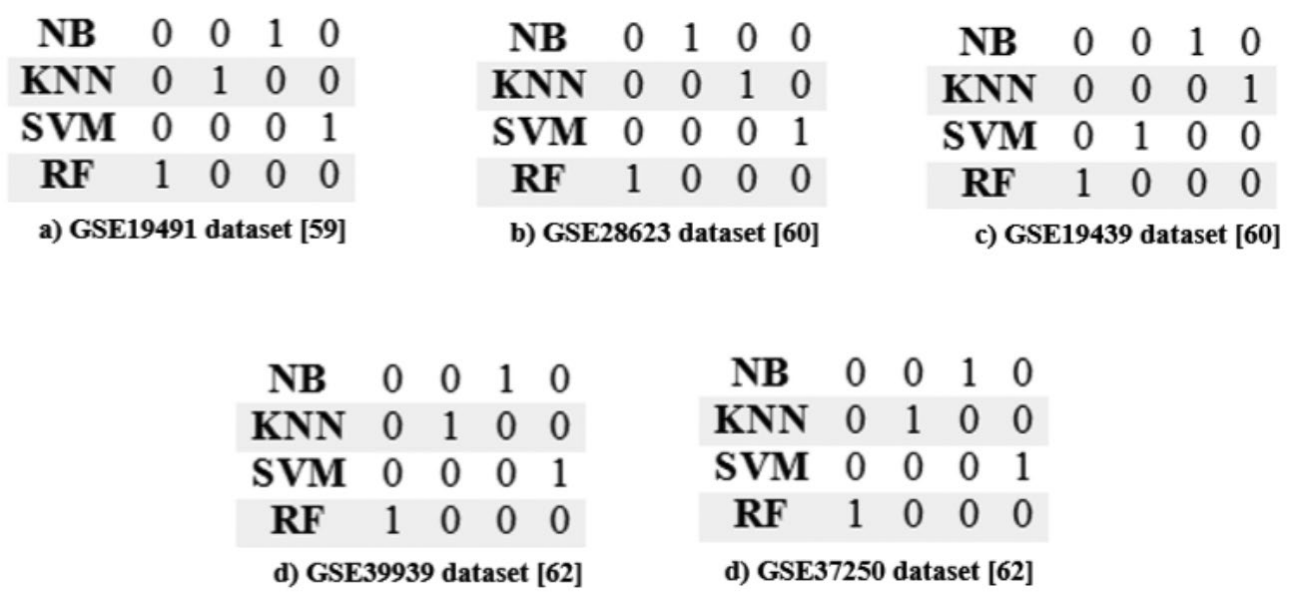
Rank matrix obtained by Linear Assignment method on the five microarray datasets

The Linear Assignment method can rank the classifiers and find the best classifier. In this study, the accuracy value obtained from applying five classifiers on genes has been used to select the best classifiers with the help of Linear Assignment. This value is used as the main questionnaire in Linear Assignment method that should be normalized, the rank matrix is created according to the definitions and equations presented in Sect. 2.3.1. In this study, we have five classifiers and ten feature selection criteria; the goal here is to find the best combination of classifiers and feature selection criteria. The rank matrix for five datasets is shown in Fig. 4. In this study, the correlation coefficient, MIM [11], MIFS [11] are increasing criterion, and the entropy is decreasing criterion.

According to Fig. 4, the best classifiers are RF, KNN, NB and SVM, respectively. The rows in these matrixes correspond to NB, KNN, SVM, and RF respectively. The numbers in this matrix indicate the significance of each classifier in a peer-to-peer way. For example, the presence of 1 in the (4, 1) entry of matrix (the first column and the fourth row) for all of the datasets, indicates the superiority of the RF classifier.

Hyperparameters in classifiers such as NB, KNN, SVM, and RF are determined before the training process, directly influencing the performance and accuracy of the algorithms. The hyperparameters setting in the applied classifiers are as follows:

- **RF**: Tree depth = 9, Number of trees = 100, Criterion = Entropy.
- **NB**: NB does not have traditional hyperparameters to tune.
- **SVM**: Kernel Function = RBF, data normalization using mean and variance of features.
- KNN: Number of neighbors = 5, Distance = minKowski, data normalization using mean and variance of features.

#### Data fusion at the decision level

In this section, the data fusion at the decision level for four classifiers NB, SVM, KNN and RF is evaluated. For each of the mentioned classifiers, the confusion matrix has been used. Four classifiers have been applied to 500 best-shared genes in Step 1. The results have been combined using Yager’s theory to improve the classification accuracy and achieve more reliable results. Fusing RF and KNN classifiers can help us achieve the goal.

#### Using wrapper feature selection method, SFFS

By applying SFFS on the selected genes of the genes selected in Step 4, the best features regarding the fusion of RF and KNN classifiers for each data set were selected. The results are shown in Table 3. After applying 10 feature selection methods on each dataset and selecting the top 500 genes based on classifier performance, the selected top genes are fed into the IDE algorithm to identify the best genes according to this algorithm. The top genes chosen based on the IDE algorithm are further evaluated using the SFFS method to determine the best genes according to this approach. The SFFS method determines the suitable features (genes) to be used to achieve the desired results. Figures related to SFFS are presented in Appendix D.

**Table 3:** SFFS statistical report

	GSE 19439[70]		GSE 19491[69]		GSE 39939[72]		GSE 37250[72]		GSE 28623[70]	
	Average	SD	Average	SD	Average	SD	Average	SD	Average	SD
Ave	0.87	0.15	60.43	2.53	86.17	0.17	97,86	0.01	95,86	0.03
Entropy	91.07	0.06	78.96	0.28	88.9	0.12	77.9	0.98	82.33	0.22
Correlation coefficient	75	4.23	71.67	0.40	88.86	0.15	77.9	0.98	0.80	0.13
FDR (Fisher’s discriminant ratio) [12]	1	0	76.06	3.91	0.80	18.97	71.67	1.48	67.07	9.02
ANOVA (analysis of variance) [13–15]	0.80	0.18	0.87	0.33	0.87	0.13	71.67	1.48	67.07	9.02
MIM (Mutual Information maximization) [11]	97,86	0.01	61	4.49	87.86	0.11	88.33	0.11	95,86	0.05
JMI (Joint Mutual Information) [11]	99.66	0.02	98.75	0.02	88.86	0.11	1	0	97,86	0.03
CMIM (Conditional Mutual Info Maximization) [11]	93.33	0.04	98.75	0.02	98.75	4.12	97,86	0.01	97,86	0.01
Gene score	1	0	90	0.17	0.87	0.12	71.67	1.48	68.07	8.02
MIFS(Mutual Information feature selection) [11]	0.99	0.05	98.75	0.01	98.75	0.01	97,86	0.01	97,86	0.01

The statistical report of the results, which contains the average and standard deviation (SD) of the importance of features (genes), is shown in Table 3.

#### PPI

**Fig. 5:**
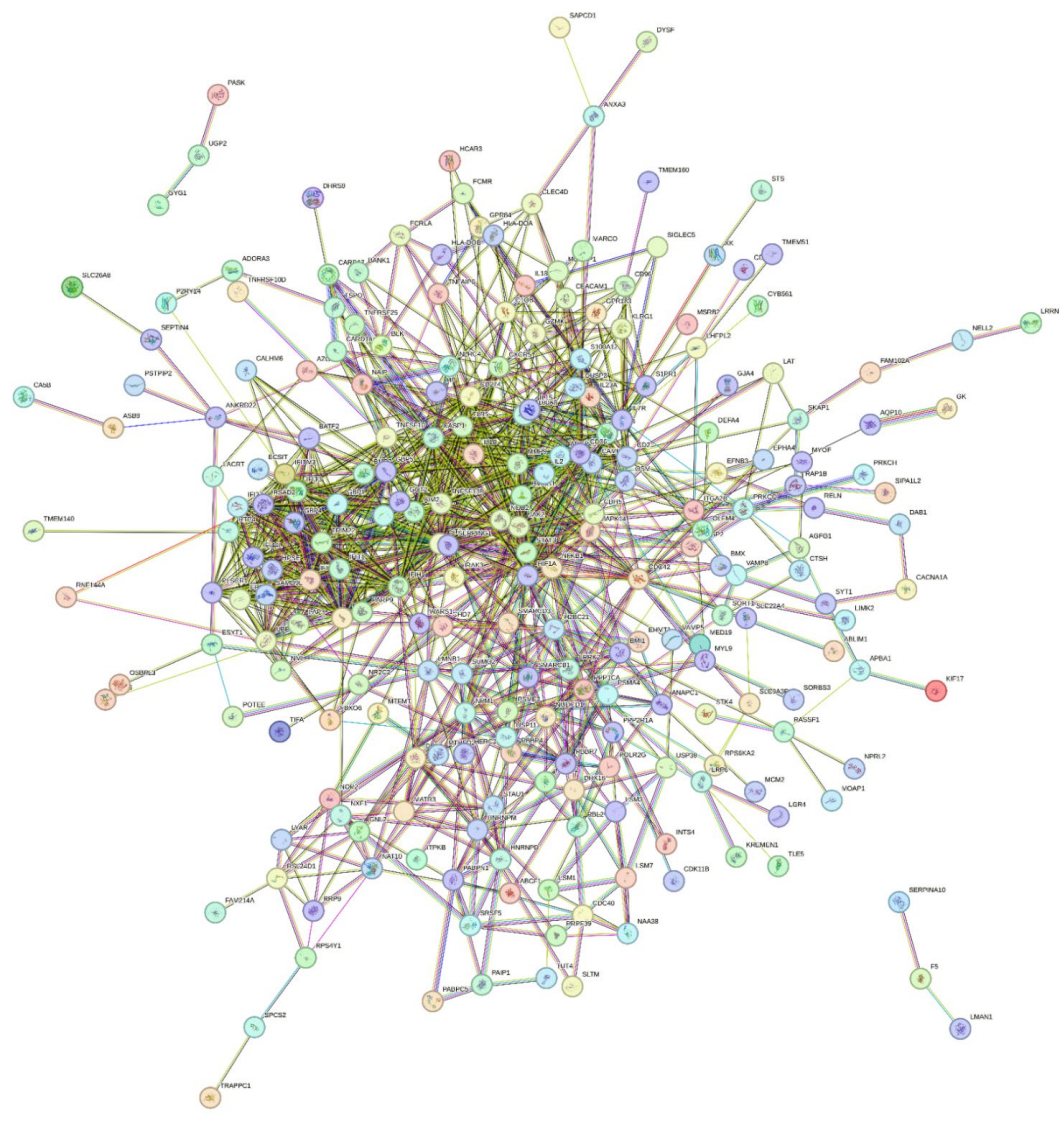
PPI network for the products (proteins) of the selected genes. The graph is generated using data from our method and visualized with STRING database [73]

PPI network has been used to prepare a communication table between features (genes) presented in Table 4. String database was used to investigate protein interactions (string-db.org) [73] and the PPI network presented in Table 4 has been used to prepare a communication table between genes. The results obtained are shown in Fig. 5. The network nodes represent proteins that are the products of the selected genes. According to Fig. 5 there are isolated nodes in the PPI network, which should be removed and not be ranked by the DEMATEL method. After removing the individual nodes, the correlation table was designed for PPIs. This table is presented in Appendix E.

**Table 4:** Superiority vector (R + C) and communication vector (R-C) for features of datasets

Gene number	Features (genes name)	*R* + C	*R*-C	Gene number	Features (genes name)	*R* + C	*R*-C
1	CA5B	0.004502	-0.0045	119	USP39	0.173723	0.0001
2	OSBPL3	0.009115	0	120	GPR84	0.176208	0
3	DYSF	0.009205	0	121	ANAPC1	0.179701	0
4	SAPCD1	0.009219	0	122	NAA38	0.180814	0.0001
5	SERPINA10	0.009219	0	123	MYL9	0.181114	0
6	DHRS9	0.009605	0	124	LSM1	0.181612	0.0001
7	PSTPIP2	0.009617	0	125	LRP6	0.188212	0.0001
8	MCM2	0.009713	0	126	GPR183	0.188507	0
9	TLE5	0.009822	0	127	STAU1	0.189121	0.0001
10	TMEM160	0.010122	0	128	CDC40	0.191804	0
11	TMEM140	0.014922	0	129	CLEC4D	0.193204	0
12	ART5	0.016201	-0.0001	130	PRKCQ	0.200717	0
13	TIFA	0.017921	0	131	RAP1B	0.201117	0
14	SLC26A8	0.01812	0	132	MCEMP1	0.209613	-0.0002
15	TRAPPC1	0.018123	0	133	VAMP8	0.210923	0
16	TMEM51	0.018422	0	134	CARD17	0.213703	0
17	PRKCH	0.019617	0	135	TNFAIP6	0.216322	0.0002
18	RNF144A	0.022618	0.0081	136	NXF1	0.220015	0.0004
19	ASB9	0.023801	0.0045	137	MATR3	0.232013	0.0166
20	POTEE	0.026116	0.0046	138	FCRLA	0.233507	0.0003
21	CDK11B	0.026804	-0.0053	139	C1QB	0.243402	-0.0001
22	LRRN3	0.027412	0	140	LYAR	0.255612	0
23	SPCS2	0.027521	0	141	CD96	0.265003	0.0005
24	SEPTIN4	0.027719	0	142	DDX1	0.266705	0.0002
25	NPRL2	0.028815	0	143	RRP9	0.266818	0
26	SORBS3	0.02922	0	144	DHX16	0.274905	0.0001
27	MYOF	0.030314	0	145	SRSF5	0.277221	-0.0001
28	ABCF1	0.0308	0	146	BLK	0.281702	0.0002
29	FAM214A	0.031006	0	147	LSM7	0.287112	0.0001
30	ITPKB	0.031111	0	148	IL18RAP	0.28791	0
31	SLTM	0.03232	0	149	GOT2	0.300307	-0.0002
32	GK	0.036607	0	150	CARD16	0.300503	0
33	MSRB2	0.036613	0.0001	151	RSL24D1	0.314218	0
34	ADORA3	0.037	0	152	PSME3	0.322117	0
35	LACRT	0.037011	0	153	PABPN1	0.325816	-0.0001
36	STS	0.037121	0	154	S1PR1	0.326119	0.0002
37	CTSH	0.038504	0	155	NAT10	0.341314	0
38	TUT4	0.038723	0	156	GZMK	0.347408	-0.0006
39	LGR4	0.039111	0	157	LSM3	0.350612	0.0056
40	CYB561	0.039204	0	158	ITGA2B	0.35441	-0.0002
41	LIMK2	0.039312	0	159	HNRNPD	0.359809	0.0001
42	P2RY14	0.041816	0.0001	160	NOP2	0.360215	0
43	HPSE	0.043009	0	161	ANGPT1	0.367601	0.0001
44	ESYT1	0.043506	-0.0045	162	POLR2G	0.369416	-0.0001
45	GJA4	0.044707	0	163	NUDCD1	0.369815	0.0003
46	NELL2	0.045514	0	164	WARS1	0.370624	0
47	LMAN1	0.045912	0	165	GNL2	0.385807	-0.0002
48	KIF17	0.046511	0	166	IRAK3	0.39731	0.0001
49	GYG1	0.046708	0	167	PPP2R1A	0.403717	0.0045
50	PASK	0.046716	0	168	LMNB1	0.411512	0.0002
51	AQP10	0.046901	0	169	SMARCB1	0.41962	-0.0001
52	MOAP1	0.048013	0	170	PSMA4	0.422817	0.0001
53	KREMEN1	0.048811	0	171	KLRG1	0.423611	-0.0273
54	SIPA1L2	0.04912	0	172	NAIP	0.450914	0.0001
55	HCAR3	0.051508	0	173	RBBP4	0.461018	-0.0004
56	ANXA3	0.052001	0	174	RBBP7	0.471718	-0.0005
57	F5	0.054606	0	175	NMI	0.476114	-0.0003
58	DAB1	0.056205	0	176	BMI1	0.487802	-0.0003
59	FAM102A	0.056606	0	177	LRRK2	0.488612	0.0001
60	CACNA1A	0.057202	0	178	PPP1CA	0.499216	0.0001
61	NR2C2	0.059615	0	179	CDH5	0.502304	0
62	CD2AP	0.059703	0	180	IL23A	0.50751	0.0005
63	LHFPL2	0.060911	0	181	SUMO2	0.510521	-0.015
64	RPS6KA2	0.063318	0	182	HNRNPM	0.520209	-0.0048
65	ABLIM1	0.0654	0	183	HP	0.523909	-0.0002
66	SIGLEC5	0.067419	-0.0001	184	S100A8	0.529319	-0.0001
67	TNFRSF10D	0.067622	-0.0005	185	SERPING1	0.547019	0.0002
68	LAT	0.068711	0	186	OSM	0.624015	0.0006
69	XK	0.075524	0	187	BATF2	0.673702	0.0001
70	MTHFD2	0.080914	0	188	CD2	0.676103	-0.0001
71	HERC2	0.081008	-0.0003	189	S100A12	0.717119	-0.0001
72	MARCO	0.082513	-0.0001	190	LAP3	0.720211	-0.0004
73	APBA1	0.084101	0	191	CXCR5	0.728404	0.0006
74	SLC9A3R1	0.08612	0	192	NLRC4	0.730614	0.0001
75	CHD7	0.086704	-0/0001	193	CAMP	0.736503	0.006
76	DUSP2	0.088705	0	194	H2BC21	0.753408	-0.0068
77	SLC22A4	0.08962	0	195	NPM1	0.898915	-0.0001
78	EPHA4	0.090206	0	196	CYBB	0.923505	0.0001
79	UGP2	0.091423	0	197	AIM2	0.924901	0.0004
80	MED19	0.092913	-0.0001	198	CDC42	0.930904	0.0002
81	ECSIT	0.094905	0	199	MAPK14	0.940813	0.0002
82	PSMG2	0.096417	0	200	TLR5	0.956622	0.0006
83	AZGP1	0.097102	0	201	PLSCR1	0.981316	-0.0232
84	BMP7	0.098802	0	202	CD36	0.989803	-0.0088
85	PABPC5	0.099416	0	203	TNFSF10	1.027622	-0.0213
86	VAMP5	0.101223	0	204	IFI27	1.053509	-0.0002
87	FCMR	0.103106	0	205	IL15	1.15491	0.0216
88	HLA-DOA	0.104608	0	206	NOD2	1.179515	0.0004
89	INTS4	0.10651	0.0001	207	HMGB1	1.199808	-0.0077
90	RELN	0.107218	0	208	IL7R	1.20291	0.0009
91	STK4	0.109521	-0.0002	209	HIF1A	1.229508	0.0005
92	FBXO6	0.112106	-0.0001	210	UBE2L6	1.246923	-0.0005
93	SORT1	0.11362	0	211	TRIM22	1.257323	-0.0005
94	ADM	0.1142	0	212	IFITM3	1.25771	0.0178
95	AGFG1	0.114701	0	213	GBP2	1.263307	0.0143
96	USP11	0.116023	0	214	TNFSF13B	1.266522	0.0006
97	BANK1	0.118402	0	215	CD274	1.266603	0.0302
98	SMARCD3	0.11842	-0.0001	216	GBP4	1.304607	0.0004
99	OLFM4	0.120415	0	217	RTP4	1.327519	-0.0004
100	PRPF39	0.123017	0	218	SAMD9L	1.398319	-0.0002
101	BMX	0.126702	0.0002	219	EPSTI1	1.420806	0.0194
102	PAIP1	0.129416	0	220	IL2	1.49501	0.0007
103	MTFMT	0.130913	0	221	MMP9	1.503013	0.0009
104	RPS4Y1	0.132518	0	222	IFI44L	1.508909	-0.0005
105	DUSP3	0.132605	0	223	JAK2	1.533711	0.0078
106	DEFA4	0.132705	0.0001	224	IFIT1	1.608909	-0.0003
107	CEACAM1	0.141204	-0.0147	225	IFIT2	1.617109	-0.0001
108	SKAP1	0.14522	0	226	GBP5	1.629007	0.0008
109	SYT1	0.151121	0	227	PARP9	1.629116	-0.0289
110	RASSF1	0.151517	0	228	RSAD2	1.649518	-0.0049
111	TSPO	0.152923	0	229	CASP1	1.685103	0.0005
112	RBL2	0.154618	0	230	IFIT3	1.727409	-0.0003
113	ANKRD22	0.160901	0	231	STAT3	1.916021	0.0078
114	EFNB3	0.162306	0.0001	232	NFKB1	2.019114	0.001
115	EHMT1	0.164606	-0.0001	233	GBP1	2.028607	0.0004
116	TNFRSF25	0.164922	-0.0005	234	IFIH1	2.307309	0.0002
117	CALHM6	0.168703	0	235	IL1B	3.01331	0.0014
118	HLA-DOB	0.173008	0.0001	236	STAT1	3.154521	0.0012

#### Using DEMATEL method for weighing features

Here, the PPI network is used to obtain the weight matrix of the DEMATEL method. The superior genes selected by feature selection methods (shown in Fig. 5) are re-ranked by DEMATEL method.

The communication table shown in Appendix E has been used in DEMATEL method to re-rank the genes in Table 4. Therefore, in this study, a new feature ranking approach is introduced in which the PPI network is used to obtain the weight matrix of the DEMATEL method. The results are given in Table 4. Finally, 26 genes are introduced that can be the most discriminative.

In Table 4, genes with negative R-C values have the lowest importance, while genes with the highest R + C values indicate greater significance in diagnosing different types of tuberculosis. More information of the gene interactions in Table 5 is provided in Appendix E.

Table 4 shows that the CYBB, AIM2, CDC42, MAPK14, TLR5, IL15, NOD2, IL7R, HIF1A, IFITM3, GBP2, TNFSF13B, CD274, GBP4, EPSTI1, IL2, MMP9, JAK2, GBP5, CASP1, STAT3, NFKB1, GBP1, IFIH1, IL1B and STAT1genes are more important and can be used as biomarkers to identify types of tuberculosis.

#### Comparison of the present study with several related studies

**Fig. 6:**
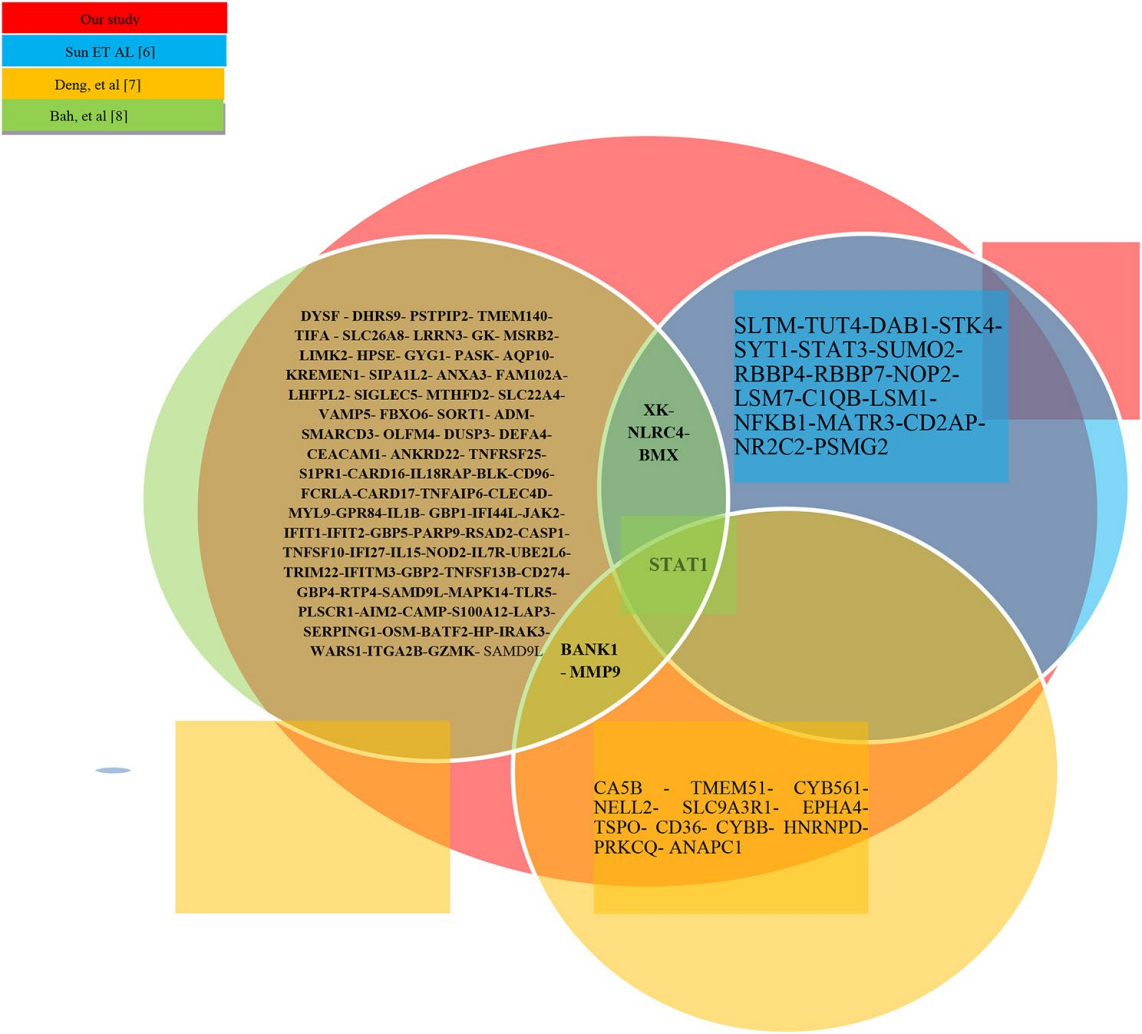
Venn diagram of common genes

Figure 6 shows that the results obtained from the present study share about 70% with previous studies [6–8]. Finally, the classifiers RF and KNN were applied on 26 introduced genes.

In this study, a combination of PPI and DEMATEL methods has been utilized, where the weights obtained from PPI were used to construct the DEMATEL ranking matrix. While this approach may increase time complexity, using appropriate feature selection methods alongside can resolve this issue, which has not been addressed in previous studies [6–8, 19].

Previous studies [6, 19] did not employ feature selection methods and directly used PPI. The direct utilization of PPI leads to computational complexity and increases the likelihood of errors when feature selection methods are not employed alongside. Data fusion at the feature and decision levels has led to the identification of top genes and suitable classifier combinations that were not utilized in prior research [6–8, 19]. Ultimately, employing these methods has enabled the discovery of latent and ATB genes. Previous studies have not utilized PPI. Considering that PPI can deeply investigate gene interactions and uncover relationships between them, its usage seems essential, despite its absence in prior studies [7, 8].

The time complexity of feature selection methods, PPI, and DEMATEL are analyzed in the following:

##### Feature selection methods

Depending on the algorithm used, these methods can have different time complexities, typically. Simple methods like filters generally have linear time complexity, where n denotes the number of features or samples. More complex methods like wrapper methods, which use machine learning models to evaluate features, may have higher time complexities, like, where is the number of features, is the number of samples, and is the number of model iterations. The process of SFFS involves iteratively adding and removing features in a dataset. Adding features requires training the model and evaluating its performance for each feature. The number of steps depends on the total number of features and dataset size, typically requiring steps per feature added. After adding features, unnecessary ones are removed similarly. Overall, SFFS’s time complexity is (*O*(*n*^2^)) due to the training and evaluation needed at each stage.

##### PPI

Time complexity in analyzing PPI networks depends on the size of the network and the analytical complexity. For instance, using graph-based algorithms for analyzing protein networks may result in a time complexity of where represents the number of nodes (proteins) and represents the number of edges (interactions) in the graph.

##### DEMATEL

DEMATEL, used to analyze relationships between variables, has a cubic time complexity (*O*(*n*^3^)), where n is the number of variables.

In Table 5, the results of these classifiers on the 26 introduced genes, in terms of fusion of classifiers of RF and KNN in Yager’s theory, have been compared with the results of former studies [7]. The fusion of RF and KNN classifiers has achieved the highest accuracy. The results are shown in Table 5. The weight of the classifiers is regarded as w = 0.98 for RF, w = 0.95 for KNN, w = 0.92 for NB, and w = 0.90 for SVM. In Yager’s theory, w = 0.90 means that a 0.10 probability of error or lack of awareness is considered for the classifier.

**Table 5:** The sensibility, specificity, and accuracy of data fusion using Yager’s theory

The classifiers fused at the decision level	Sensibility	Specificity	Accuracy
RF and NB	0.98	0.91	0.86
RF and SVM	0.98	0.43	0.67
RF and KNN	0.95	0.88	0.92
NB and SVM	0.98	0.10	0.36
NB and KNN	0.92	0.78	0.60
SVM and KNN	0.92	0.22	0.41
RF and NB and SVM	0.99	0.005	0.40
RF and NB and KNN	0.97	0.93	0.87
NB and SVM and KNN	0.94	0.39	0.48
SVM and KNN and RF	0.92	0.22	0.41
RF and NB and KNN and SVM	0.99	0.81	0.71

In this study, RF-KNN fusion, applied to the output of best feature selection criteria (i.e., correlation coefficient, MIM, MIFS and entropy) achieved the highest accuracy in classifying LTBI expression data (accuracy of **0.92** on the test data(GSE 19444 [71]). The results are shown in Table 6.

**Table 6:** The sensibility, specificity, and accuracy of applying classifiers on 26 introduced genes compared to the results of former studies

	Classifiers	Our study	Study of Deng, et al. [7]
Sensibility	RF	0.91	-
	NB	0.86	-
	KNN	0.59	
	SVM	0.95	0.907
	RF and KNN	0.95	-
Specificity	RF	0.83	-
	NB	0.58	-
	KNN	0.5	
	SVM	0	0.913
	RF and KNN	0.88	-
Accuracy	RF	0.94	-
	NB	0.49	-
	KNN	0.47	
	SVM	0.38	0.911
	RF and KNN	0.92	-

**Fig. 7:**
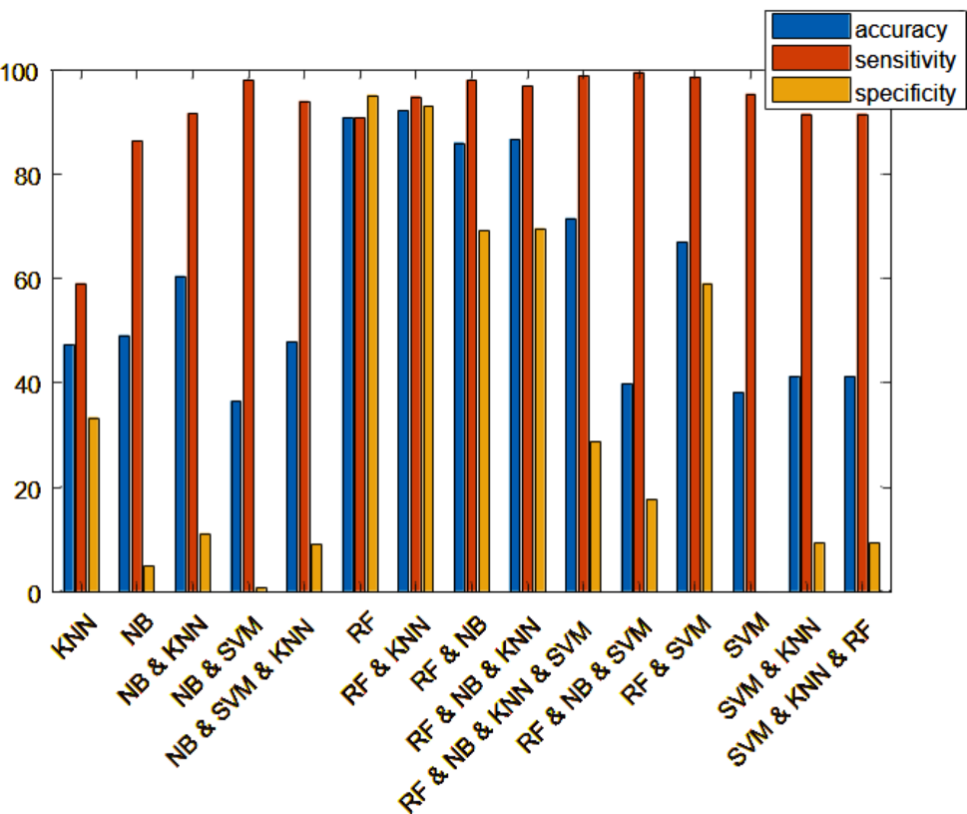
The sensibility, specificity, and accuracy of applying confusion classifiers on 26 introduced genes

The sensibility, specificity, and accuracy of applying confusion classifiers on 26 introduced genes are shown in Fig. 7 (highest accuracy value of the fusion of RF and KNN classifiers on the test data (GSE 19444 [71]).

Common genes of the current study, along with the research of Sun et al. [6] and Bah, et al. [8] introduced in the Venn diagram of Fig. 6, were examined to introduce the distinguishing genes between the latent and active states of TB on the GSE 19444 [71] (Data set test). The result is presented in Table 7.

**Table 7:** Distinguishing genes between the latent and active states of TB

latent tuberculosis	ATB
NFKB1-MATR3- CD36-CD2AP-PSMG2-TNFSF13B-IL23A-DUSP2-STAT1-SLC9A3R1-RELN-NR2C2 -NLRC4-CA5B-PRKCQ-TNFAIP6-ANKRD22- DAB1	TNFAIP6-GBP4-GBP5-SAMD9L-SERPING1-BANK1-CYBB-HNRNPD -HLA-DOB-TSPO

Hierarchical clustering and accumulative clustering were used for further investigation and to obtain more assurance of the introduced genes that differentiate LTBI and ATB states.

The accumulative clustering indicates whether the genes located in one cluster and one data set are located similarly in the same cluster in another data set or not. This procedure is applied to the GSE37250 [72] and the GSE39939 [72] data sets.

**Fig. 8:**
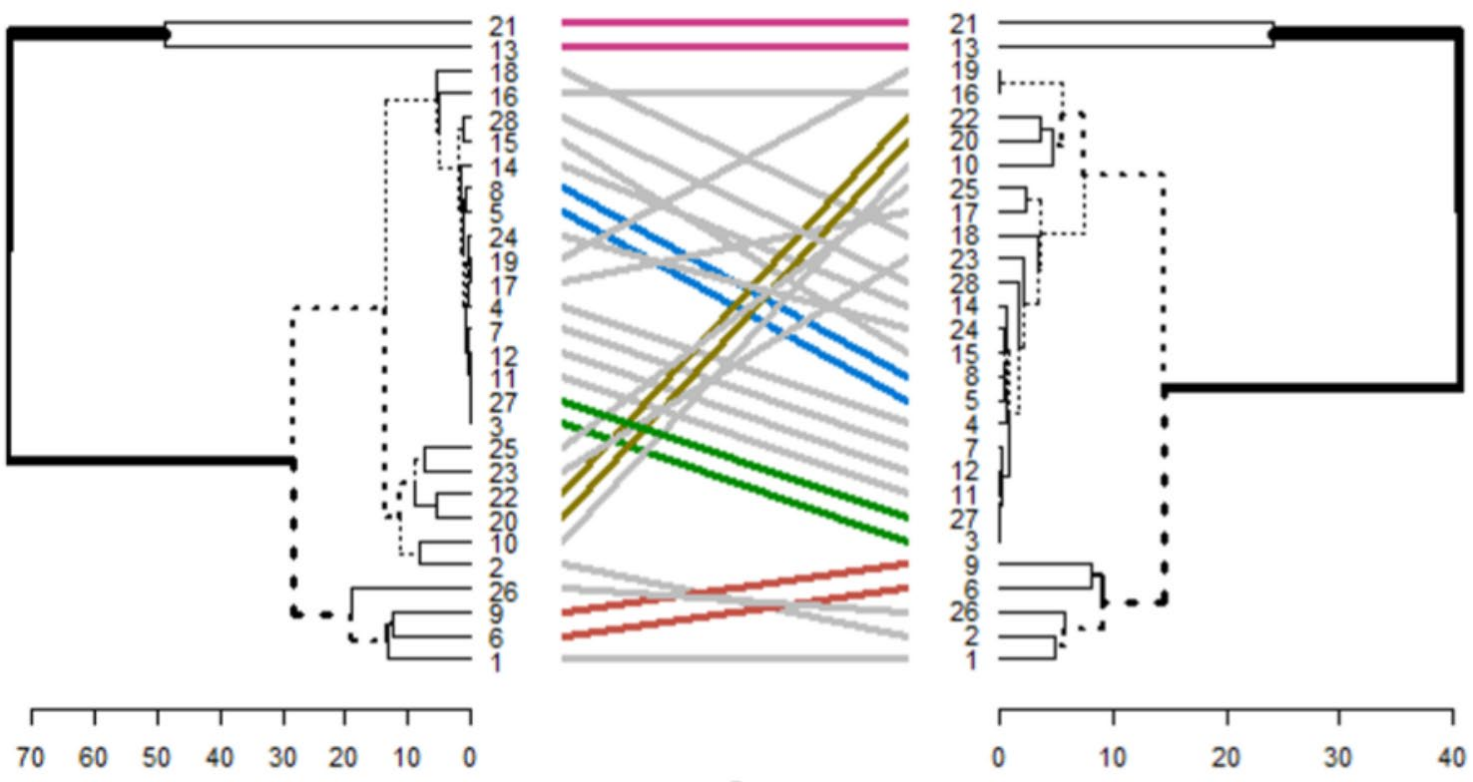
Accumulative clustering for genes in Table 8

Accumulative clustering for genes in Table 8 is shown in Fig. 8.

**Fig. 9:**
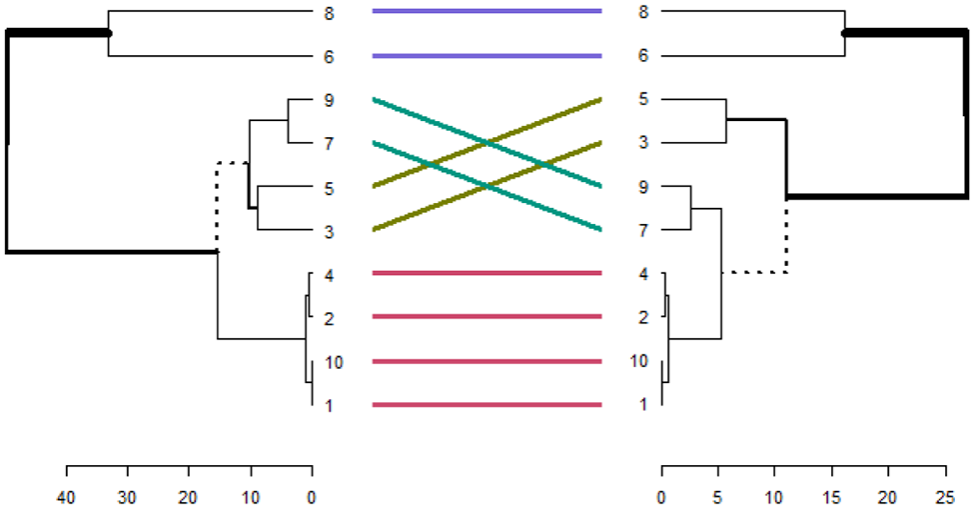
Accumulative clustering for colored significant relations shown in Fig. 8 for GSE37250 [72] and the GSE39939 [72] data sets

The colored significant relations shown in Fig. 8 are shown and analyzed separately in Fig. 9. In Fig. 8, the labeled gene numbers on the right side corresponds to the GSE37250 [72] dataset, and the left one corresponds to the GSE39939 [72].

Genes CD36, PSMA4, TNFSF13B, DUSP2, STAT1, TSPO, GBP4, GBP5, SAMD9L, and DAB1 correspond to labeled gene numbers 1 to 10 in Fig. 9. The accumulative clustering analyses in Fig. 9 demonstrated that our gene pairs preferred to cluster within the topological and functional modules. Common gene pairs extracted from Table 8 shows the strongest tendency in this regard. Genes 1, 2, 3, 4, 5, 6, 8, 10, which are related to the latent state of tuberculosis, were located in identical and close clusters. This activation tendency is also true for genes 7 and 9, which are the genes that activate tuberculosis. The ATB-specific genes showed higher expression values and fold changes than the LTBI-specific genes. Furthermore, the ATB-related pairs generally displayed higher expression correlations and were more activated when compared to their LTBI-related counterparts.

According to the results of Fig. 9, it was found that the pairs of genes GBP5-TSPO, STAT1-TNFSF13B, DUSP2-PSMG2, and DAB1-CD36, are the latent factors of tuberculosis, and the pair of genes SAMD9L-GBP4 are the factors of activating tuberculosis.

After applying cumulative clustering on the GSE19491 dataset [69] and GSE19444 dataset [71], we found that MATR3-NR2C2 gene pair is the cause of TB latency, and SAMD9L-GBP4 gene pair is the cause of TB activation.

The sensibility, specificity, and accuracy of applying classifiers on 10 introduced genes compared to the results of former studies [7] presented in Table 8.

**Table 8:** The sensibility, specificity, and accuracy of applying classifiers on 10 introduced genes compared to the results of former studies

	Classifiers	Our study	Study of Deng, et al. [7]
Sensibility	RF	0.93	-
	NB	0.86	-
	KNN	0.87	
	SVM	0.95	0.907
	RF and KNN	0.97	-
Specificity	RF	0.89	-
	NB	0.60	-
	KNN	0.65	
	SVM	0.45	0.913
	RF and KNN	0.90	-
Accuracy	RF	0.95	-
	NB	0.60	-
	KNN	0.72	
	SVM	0.62	0.911
	RF and KNN	0.95	-

In order to compare the results presented in this study and previous studies in the field of LTBI diagnosis, the AUC value has been approximated using the Eq. (21).

21 $${\rm{AUC}} \approx {{{\rm{Accuracy}} - 0.5} \over {0.5}}$$

The most relevant studies [20–24, 26–29] in the field of detection of TB differential genes, which were reviewed in the related work section, have been compared with the present study in Table 9 in term of AUC. The introduced genes in Table 9 are the combination of introduced genes in Step 6 and Step 8 of the proposed method. In the Step 6 of the proposed method, 26 discriminative genes are introduced and in the Step 8 of the proposed method, 10 discriminative genes are introduced. Common genes between this study and previous studies are shown in bold.

**Table 9:** Comparison of the present study with previous studies

	Methods used	Introduced differentiating genes	Used datasets	Number of introduced genes	Evaluation
Our study	Machine learning Data fusion PPI MCDM	CYBB, **AIM2**, CDC42, MAPK14, TLR5, IL15, NOD2, **IL7R**, HIF1A, IFITM3, GBP2, TNFSF13B, **CD274**, **GBP4**, EPSTI1, IL2, MMP9, JAK2, **GBP5**, **CASP1**, STAT3, NFKB1, **GBP1**, IFIH1, IL1B, CD36, PSMA4, DUSP2, **STAT1**, TSPO, **SAMD9L**, and DAB1	GSE 19444 GSE19491 GSE39939 GSE37250 GSE28623 GSE19439	32	AUC = 84% for 26 genes and 90% for 10 genes
study of Juan Zhang et al. [20]	weighted gene co expression network analysis (WGCNA) PPI	**SAMD9L**, **GBP1**, **GBP5**, and **STAT1**	GSE 19444 GSE 19443	4	AUC = 0.865
study of Wu et al. [21]	PPI	**IL7R**, TNFAIP6, KLHDC8B, IRF1, HELZ2, ADM, CALHM6, GCH1, **CD274**, CCR7, and PSTPIP2.	GSE83456 GSE19491 GSE50834	11	AUC = 0.801
study of Natarajan et al. [22]	PPI	FCGR1B, ANKRD22, CARD17, IFITM3, TNFAIP6, FCGBP, KLF12	GSE37250 GSE19491	7	AUC = ranged from 0.84 to 1
study of Liu et al. [23]	Neural network WGCNA	**AIM2**, CASP8, and NAIP	GSE39940 GSE37250	3	AUC = ranged from 0.787 to 0.946
study of Dai et al. [24]	WGCNA PPI RF	**CASP1**, TNFSF10, CASP4, CASP5, IFI16, and ATF3	GSE19491 GSE62525 GSE28623	6	AUC = greater than 0.7
study of Chen et al. [26]	WGCNA	FBXO6, ATF3, **GBP1**, **GBP4**, and **GBP5**	GSE19491 GSE98461 GSE152532	5	AUC = ranged from 0.8 to 0.9
study of Yu et al. [27]	RF SVM general linear model (GLM)	CD247, MAN1C1, FAM84B, HSZFP36, SLC16A10, DTX3, and SIRT4	GSE39939 GSE39940	7	AUC = 0.950
study of Chen et al. [28]	eXtreme Gradient Boost (XGB) general linear model (GLM) RF SVM	NFE2L2, NLRP3, FDX1, LIPT1, PDHB, MTF1, GLS, DBT, DLST, MAN1C1, DKFZP434N035, SIRT4, BPGM, and APBA2	GSE39939 GSE39940	15	AUC = 0.905
study of Wang et al. [29]	Bioinformatic methods	FOXO1, CCL2, and ITGA3	GSE37250 GSE19491 GSE28623	3	AUC = ranged from 0.827 to 0.883

To compare the current study with studies listed in Table 2 [20–24, 26–29], the following points can be highlighted:

• AUC:

In the present study, the AUC obtained is higher compared to several studies [20, 21, 24, 26, 29]. This indicates an improvement in the performance of the proposed model over previous methods.

• Number of Microarray Datasets:

In this study, the number of microarray datasets examined is greater than those used in all studies listed in Table 10 [21, 28]. This demonstrates a more comprehensive exploration of datasets, potentially providing broader applicability for our model.

• Number of Genes Introduced:

Furthermore, the number of genes introduced in the current study is fewer than those introduced in several studies listed in Table 10 [20–24, 26–29], indicating a higher discriminatory ability of the introduced genes.

Studies that solely rely on bioinformatic methods such as protein-protein interaction networks (PPIN) without using machine learning [20–22, 26, 29] may face several challenges and weaknesses such as low prediction accuracy and inability to understand biological complexities.

To investigate the contribution of each component of our proposed method on the overall performance, the ablation study is conducted. The ablation study results are presented in Table 10.

**Table 10:** The ablation study results in terms of sensibility, specificity, and accuracy (applying RF-KNN classifiers on GSE 19444)

Feature Selection and selecting 500 genes	Feature Fusion: The IDE method → Optimal Gene Combination(SFFS)	Combination PPI-DEMATEL and introduce 236 genes	Decision Fusion on 26 genes	Decision Fusion on 10 final genes	sensibility	Specificity	Accuracy
√					0.83	0.80	0.81
√	√	√			0.89	0.85	0.88
**√**	**√**	**√**	**√**		**0.95**	**0.88**	**0.92**
**√**	**√**	**√**	**√**	**√**	**0.97**	**0.90**	**0.95**

## Conclusion

The goal of this study was to minimize the number of features of tuberculosis data. The second goal is to classify tuberculosis data using a subset of genetic features obtained by the first goal. In this study, data fusion methods, MCDM, feature selection and PPI network were utilized to identify LTBI distinguishing genes from Healthy control and ATB. Filter feature selection methods and SFFS methods have been used to create more overlap of genes among studies and increase the accuracy in introducing biomarkers with greater distinguishing power. The PPI network is used to obtain the weight matrix of the MCDM method.

In the data fusion at the feature level, IDE feature selection method is applied on the top 500 genes in terms of the best classifier introduced in each feature selection criteria. Also, in the data fusion at the decision level, it is determined which classifiers should be fused to achieve better results. In this paper, a new approach based on Dempster-Shafer and Yager’s theory is proposed to fuse the effects of classifiers. Therefore, the proposed method introduces a suitable set of feature selection criteria and a suitable set of classifiers to achieve a reliable diagnosis of latent tuberculosis. According to the results, fusion of correlation coefficient, MIM [11], MIFS [11] and entropy features selection method, and the fusion of RF and KNN classifiers can be used to identify latent tuberculosis genes.

Finally, the 26 genes were selected, and some of these genes were shared with the results of previous studies [6–8]. The results of our study were able to identify more LTBI genes with higher accuracy, analyzing more datasets and providing a more limited set of genes differentiating LTBI compared to the results of studies conducted by Wang et al. [19] and Bah et al. [7]. The main weaknesses of other approaches [7, 8] is the low accuracy of previous biomarkers, lack of stability due to small overlap of genes among studies, and the lack of integration helpful information such as the PPI network. The introduced genes can be used in many applications such as disease risk prediction systems.

At the end of the current research, with the help of hierarchical clustering and accumulative clustering, the introduced genes were reanalyzed to differentiate between latent and ATB states more reliably. Additionally, several pairs of genes responsible for the activation and hiding of tuberculosis were introduced.

In the future, the proposed procedure may be extended and applied to other datasets and used some new classification and feature selection methods to diagnose tuberculosis and other diseases. In the current study, the traditional machine learning methods were applied. In future work, deep learning techniques can be applied instead of traditional machine learning methods to improve the results [74].

## Electronic supplementary material

Below is the link to the electronic supplementary material.

Supplementary Material 1

## Data Availability

The datasets used in this study were collected from the NCBI website (https://www.ncbi.nlm.nih.gov/). Part of the data, which is related to the results of the article, has been uploaded as Supplementary material and related file, and is included in the Appendix E of the paper.
