# Supplementary material for: A modified multiple-criteria decision-making approach based on a protein-protein interaction network to diagnose latent tuberculosis
Source: BMC Med Inform Decis Mak. 2024 Oct 30;24:319. doi: 10.1186/s12911-024-02668-z (PMC11523813; doi:10.1186/s12911-024-02668-z)
Supplement: Supplementary file 1 — Supplementary Material 1 [file 12911_2024_2668_MOESM1_ESM.pdf]

Appendix A. The six datasets, the number of their samples and various disease states.

Table A1 List of data sets with several samples and different disease states

GSE	Healthy control	Latent tuberculosis	Active tuberculosis	Number of features (genes)
19491[69]	133	69	89	48803
28623[70]	37	25	46	45015
19444[71]	12	21	21	48803
19439[70]	12	17	13	48791
39939[72]	-	14	79	47323
37250[72]	-	167	195	47323

The dataset included other labels, but we do not focus on them in this study.

Appendix B. Statistical criteria and filter feature selection methods

Table B1 Equations of Statistical criteria

Name	Equation
Ave	$B2 = \frac{1}{n}(\frac{\sum_{j=1}^{k} x(n)}{n})$
Entropy	$B8 = -\sum_{i,j} p(xi,yi)\log\frac{p(xi,yi)}{p(yi)}$
Correlation coefficient	$B9 = \frac{cov(x,y)}{\sigma x\, \sigma y} = \frac{E[(x-\mu x)(y-\mu y)]}{\sigma x\, \sigma y}$
FDR (Fisher's discriminant ratio) [12]	$B11 = \frac{Variance\ of\ means\ between\ the\ classes}{Average\ Variance\ of\ within\ the\ classes} = F-ratio = \frac{\sum_{j=1}^{k}(\mu j-\mu)^2}{\frac{1}{k}\sum_{j=1}^{k}\frac{1}{nj}\sum_{i=1}^{nj}(xij-\mu j)^2}$
ANOVA (analysis of variance) [13-15]	$B12 = F = \frac{MST}{MSE} = \frac{Mean\ square\ due\ to\ Treatment}{Mean\ square\ due\ to\ Erorr} \rightarrow P-value$
MI (Mutual Information) [11]	$B13 = I(Y;X) = \begin{cases} H(Y)-H(Y\vert X) \\ H(X)-H(X\vert Y) \\ H(Y)+H(X)-H(Y,X) \end{cases}$ $= \sum_{x\in X}\sum_{y\in Y} p(y,x)\log\frac{p(y,x)}{p(y)\,p(x)}$
Jmi (Joint Mutual Information) [11]	$B14 = I(X;Y) = H(X)-H(X\vert Y) = \sum_{x\in X}\sum_{y\in Y} p(xy)\log\frac{p(xy)}{p(x)p(y)}$
Cmim (Conditional Mutual Info Maximization) [11]	$B15 = I(Y;X_i\mid X_j) = \begin{cases} H(Y\vert Xj)-H(Y\vert Xi,Xj) \\ H(Xi\vert Xj)-H(Xi\vert Y,Xj) \\ I(Xi;Y\vert Xj) \end{cases}$

Appendix C. The results of step2 of the proposed method

Table C1 The accuracy for using several feature selection criteria and applying the three classifiers on the selected feature

Feature selection criteria											
Gene Expression Series (GSE)	Classifiers	ave	RMS	Kurtosis	Correlation coefficient	variance	Entropy	Skewness	mean	STD	Gen-score [16]
19491[69]	NB	87±0.1	52±0.06	88.6±0.12	88.9±0.2	37±0.002	89±0.15	89±0.11	47±0.003	47±49±0.05	88.9±0.11
	KNN	**79±0.1**	50±0.06	88.6±0.12	**86±0.2**	47±0.002	**88±0.15**	87.7±0.11	49±0.003	49±0.05	94±0.11
	SVM	79±0.1	60±0.06	78.6±0.12	99±0.1	69±0.002	98.5±0.15	97.7±0.11	39±0.003	49±0.05	86±0.11
	RF	88.9±0.1	79±0.06	99±0.1	98.9±0.1	78.9±0.002	89±0.15	99±0.1	78.9±0.003	88.9±0.05	98.9±0.11
28623[70]	NB	87±0.1	45±0.06	88.6±0.12	88.9±0.2	37±0.002	87±0.15	89±0.11	47±0.003	47±0.05	74.2±0.11
	KNN	**89±0.1**	50±0.06	88.6±0.12	**97±0.2**	47±0.002	**88±0.15**	87.7±0.11	49±0.003	49±0.05	87±0.11
	SVM	77±0.1	48±0.06	78±0.12	89±0.2	89±0.002	98.1±0.15	97.7±0.11	39±0.003	39±0.05	87.6±0.11
	RF	84.2±0.1	70±0.06	99±0.1	98.9±0.2	88.9±0.002	97.8±0.15	99±0.1	78.9±0.003	88.9±0.05	94.2±0.11
19439[70]	NB	87±0.1	59±0.06	77.6±0.12	88.9±0.2	37±0.002	88.9±0.15	89±0.11	47±0.003	47±0.05	84.2±0.11
	KNN	**89±0.1**	44±0.06	77±0.12	**86±0.2**	47±0.002	**87±0.15**	87.7±0.11	49±0.003	49±0.05	97±0.11
	SVM	79±0.1	67±0.06	97±0.12	99±0.1	77±0.002	97.4±0.15	97±0.11	39±0.003	39±0.05	86.5±0.11
	RF	88.9±0.1	86.9±0.06	99±0.1	88.9±0.2	88.9±0.002	98.9±0.1	99±0.1	78.9±0.003	88.9±0.05	98±0.11
39939[72]	NB	87±0.1	60±0.06	79±0.12	88.9±0.2	37±0.002	88.9±0.15	89±0.11	47±0.003	47±0.05	93±0.11
	KNN	**79±0.1**	48±0.06	79.2±0.12	**80±0.2**	47±0.002	**88±0.15**	87.7±0.11	49±0.003	49±0.05	96±0.11
	SVM	79±0.1	44±0.06	99.2±0.1	99±0.1	59±0.002	98.91±0.1	99.7±0.1	39±0.003	39±0.05	86±0.11
	RF	88±0.1	67±0.06	99±0.1	88.9±0.2	77.7±0.002	98.9±0.1	99±0.1	87±0.003	77.7±0.05	98.9±0.11
37250[72]	NB	87±0.1	59±0.06	89±0.12	88.9±0.2	37±0.002	88.7±0.15	89±0.11	47±0.003	47±0.05	93±0.11
	KNN	**83±0.1**	55±0.06	88.6±0.12	**85±0.2**	47±0.002	**83±0.15**	87.7±0.11	49±0.003	49±0.05	96±0.11
	SVM	78±0.1	66±0.06	78.6±0.12	88±0.2	58±0.002	90±0.15	87.7±0.11	39±0.003	39±0.05	86±0.11
	RF	88.9±0.1	70±0.06	99±0.1	88.9±0.2	77.7±0.002	98.9±0.1	99±0.1	87±0.003	77.7±0.05	98.9±0.11

Appendix D. Data fusion at the feature level, and use of the IDE method

This paper provides a summary of the use of IDE plots for selecting top genes in microarray analysis. IDE plots measure the importance of each gene in the dataset. Typically, IDE plots include axes representing gene numbers or positions on the microarray and their corresponding IDE values, which indicate their significance in the samples. Genes with higher IDE values are identified as top genes, guiding researchers on which genes to prioritize for further biological studies. IDE plots assist researchers in making informed decisions for biological investigations, thereby enhancing their understanding of their samples and gene relationships. In figures 4 and 6, rows represent features (genes), and columns depict the accuracy level of each feature. The IDE (Importance Density Estimation) plot is utilized for selecting top genes in microarray data. It illustrates the importance or significance of each gene in the dataset, aiding researchers in identifying which genes provide the most valuable information for biological studies. This

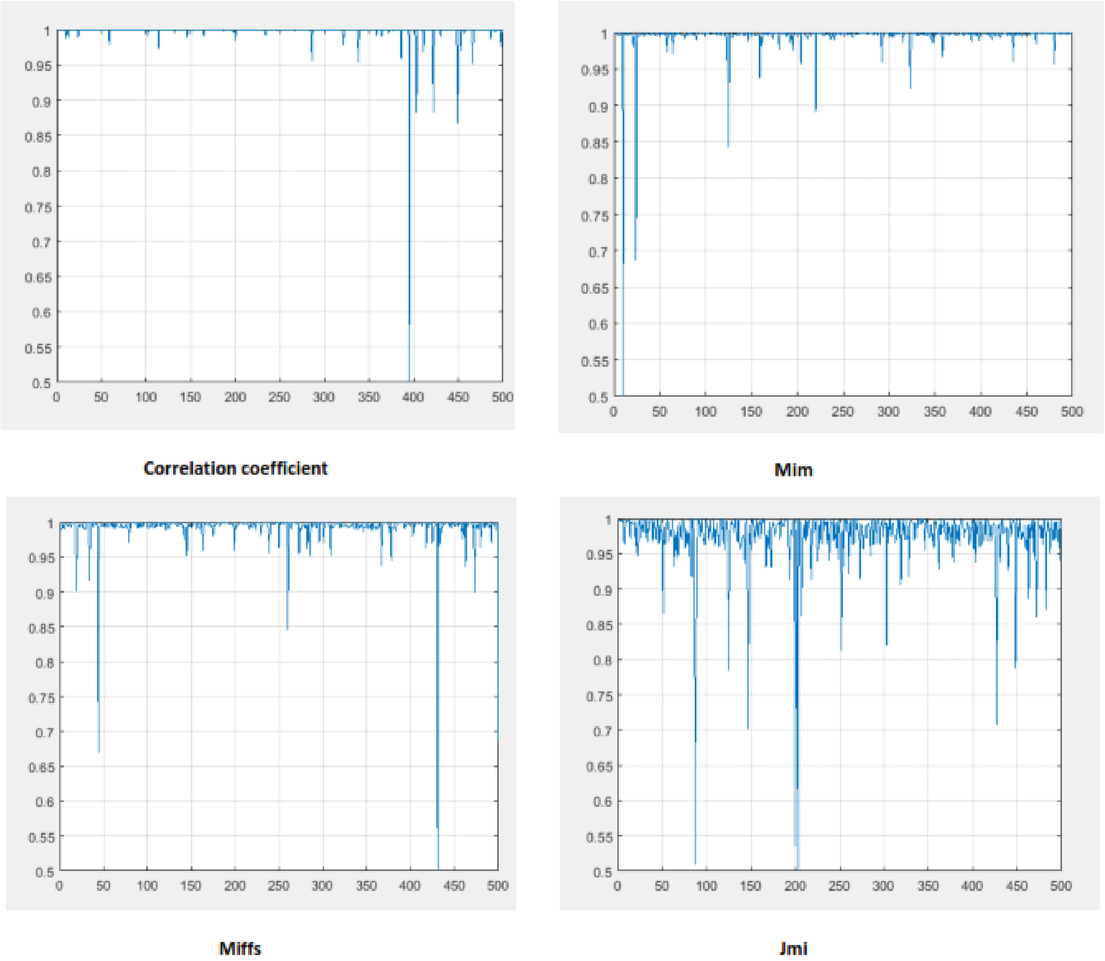

Figure 4 The importance of genes obtained by applying IDE method on the 5 microarray datasets - GSE37250 dataset [72]

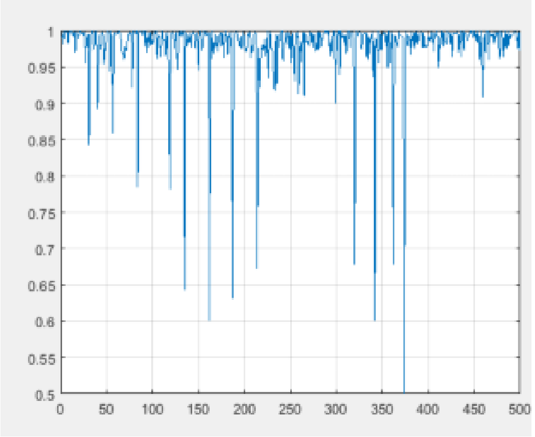

Cmim

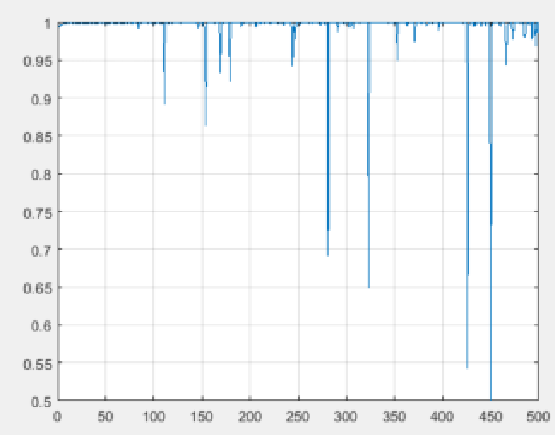

Entropy

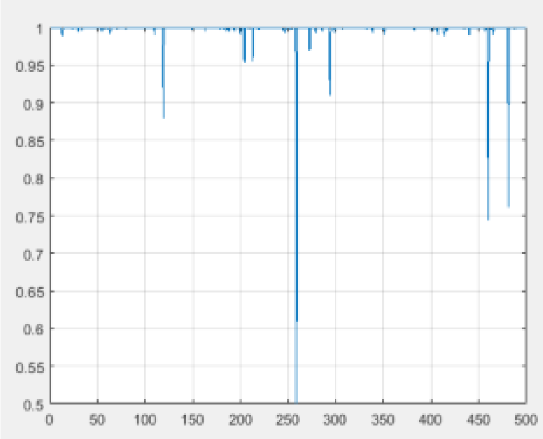

ANOVA

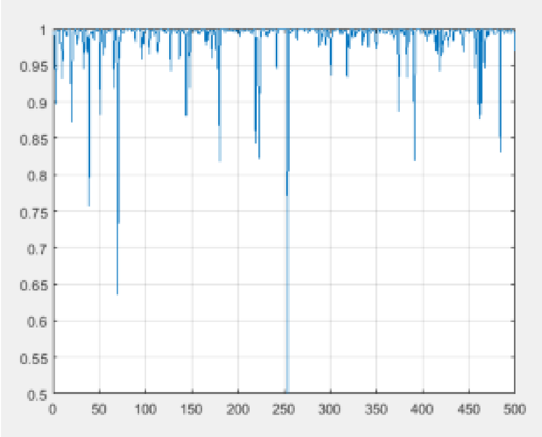

FDR

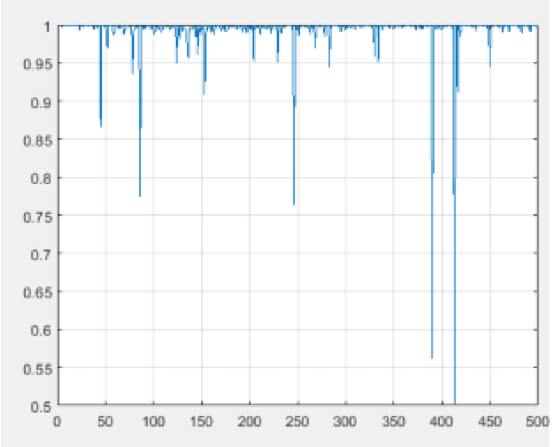

Ave

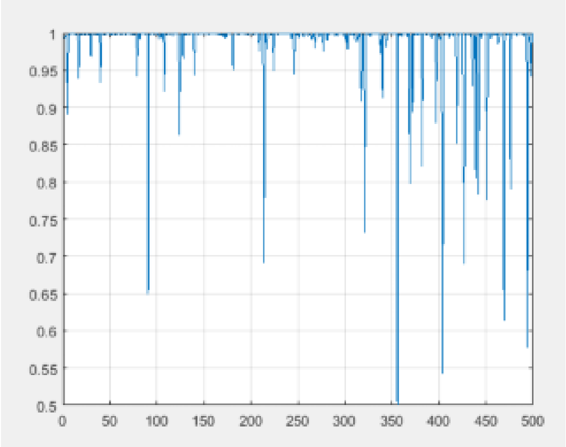

Gen-score

Figure 4 (continued) - GSE37250 dataset [72]

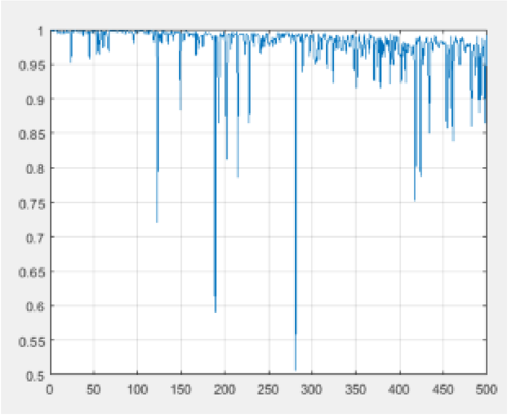

Correlation coefficient

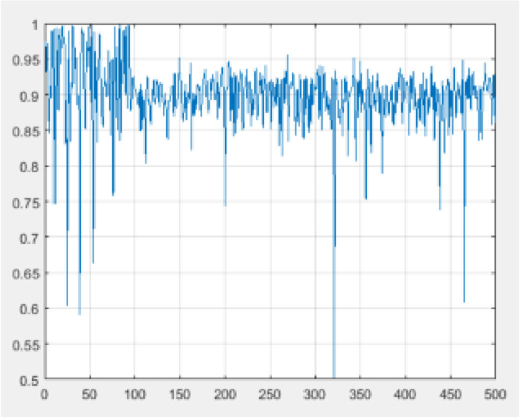

Mim

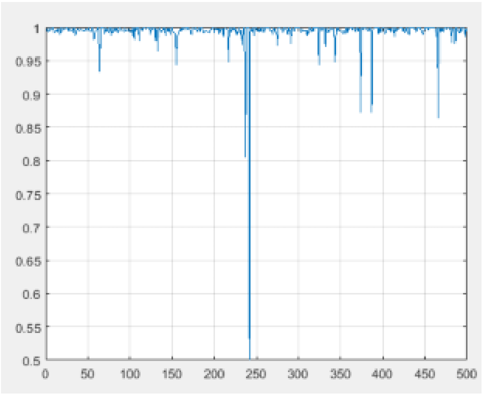

Miffs

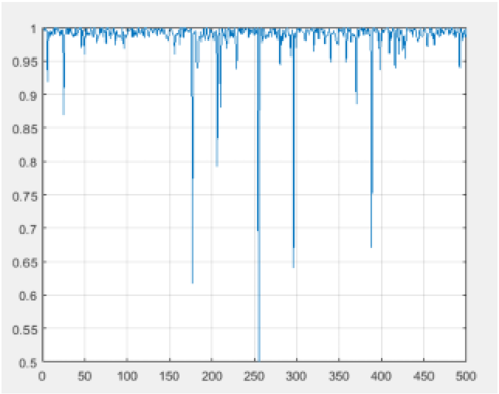

Jmi

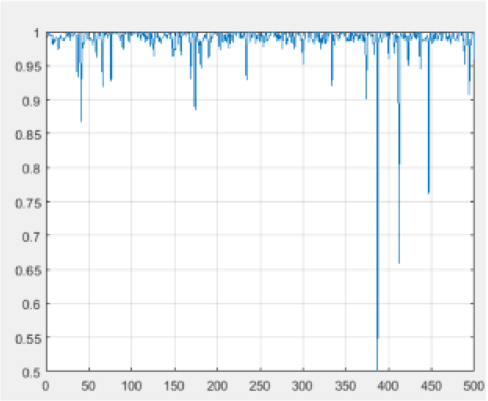

Cmim

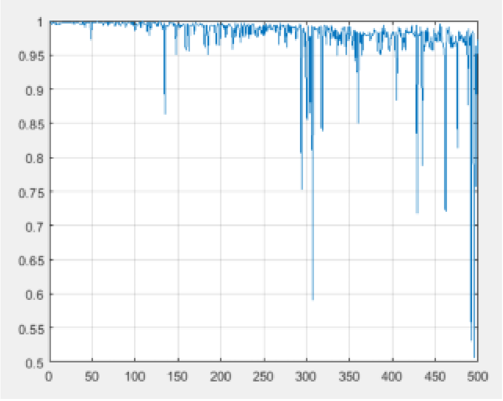

Entropy

Figure 4 (continued) - GSE28623 dataset [70]

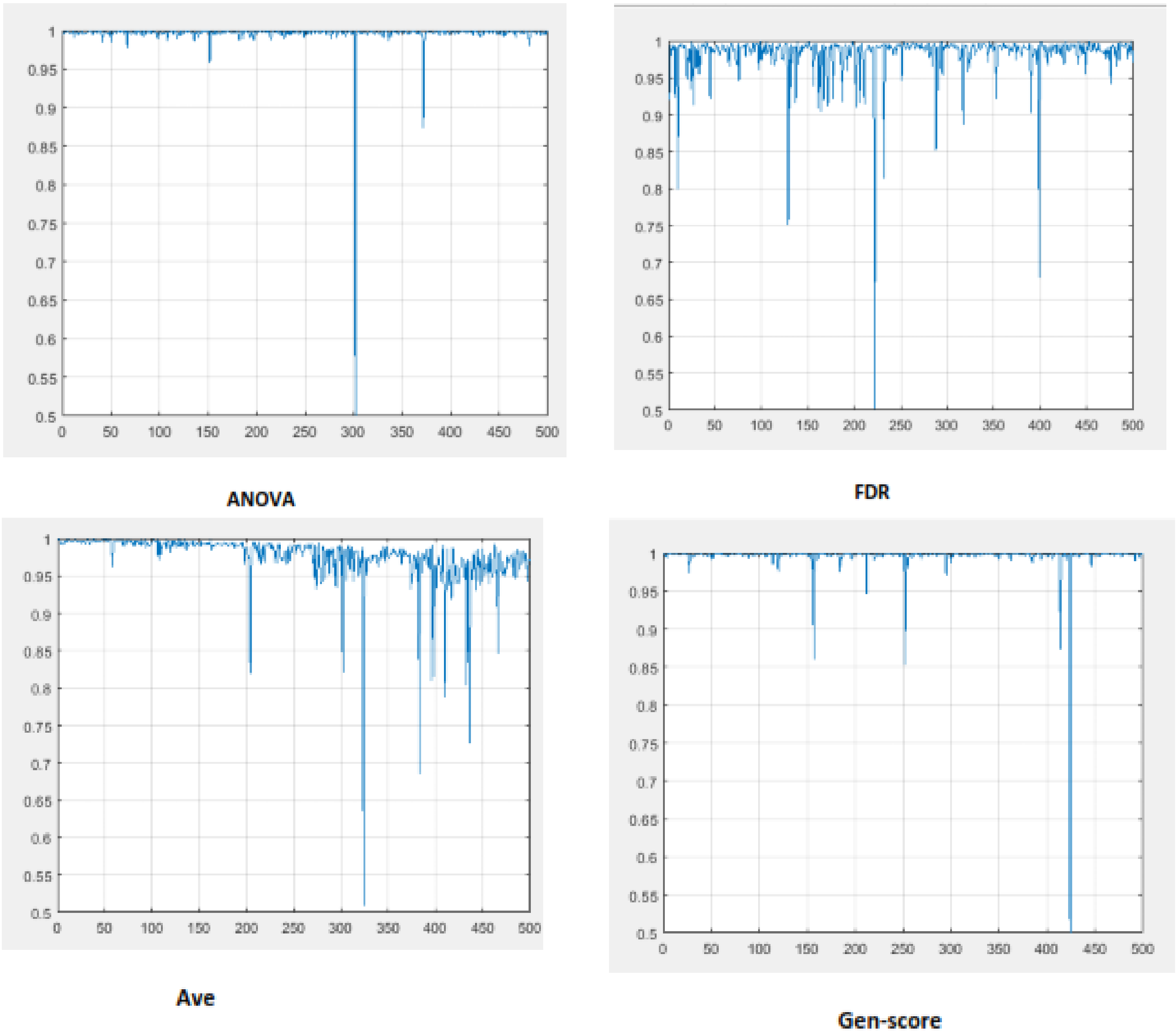

Figure 4 (continued) - GSE28623 dataset [70]

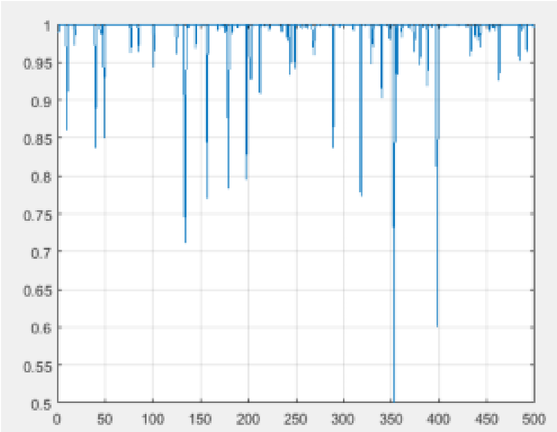

Correlation coefficient

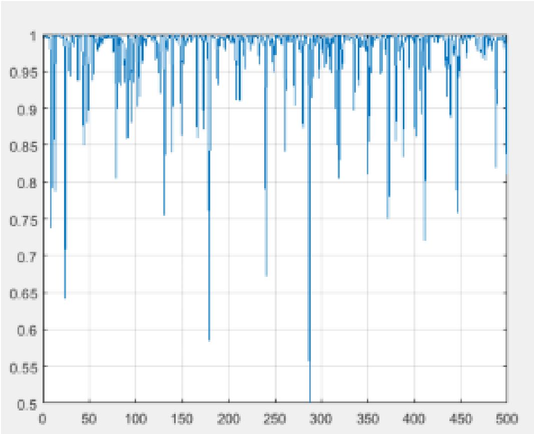

Mim

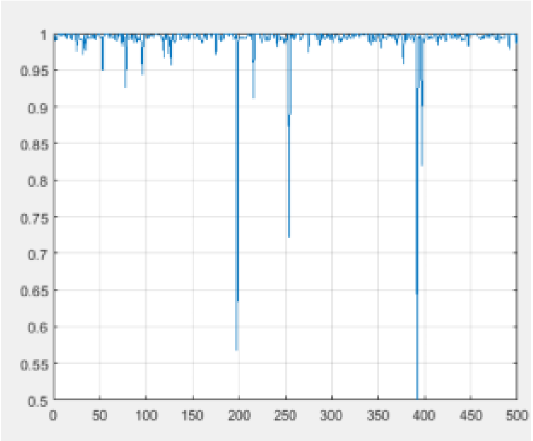

Miffs

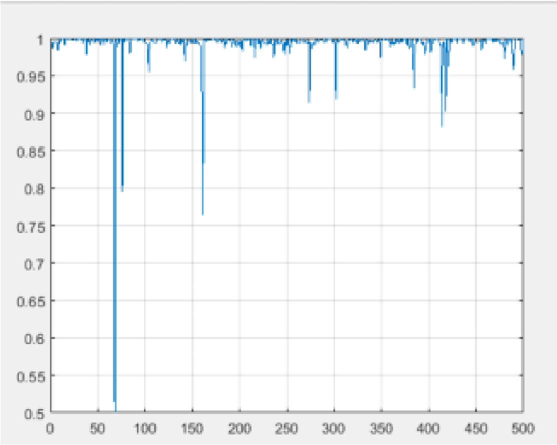

Jmi

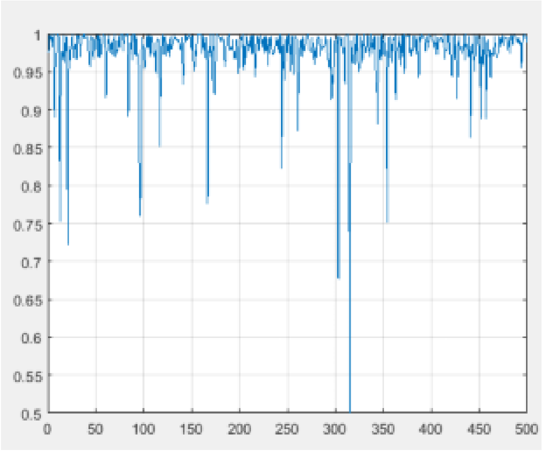

Cmim

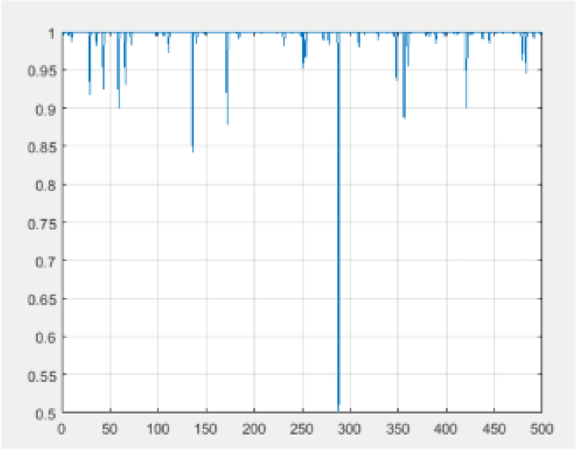

Entropy

Figure 4 (continued) - GSE19491 dataset [69]

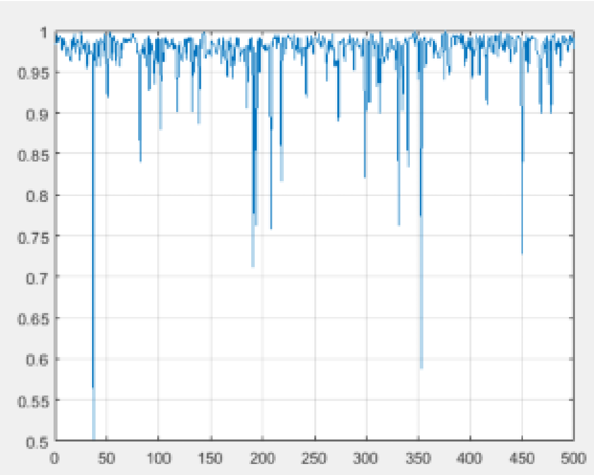

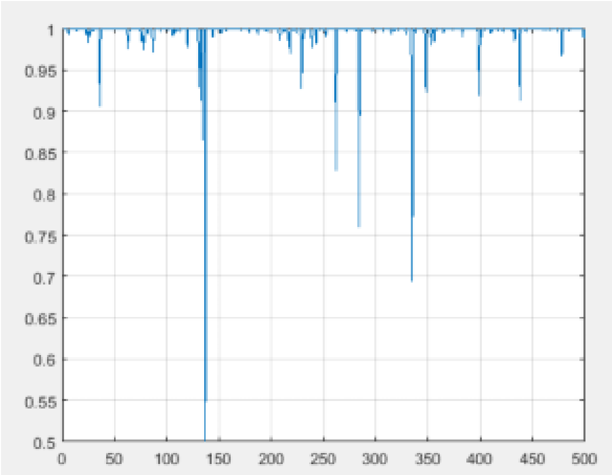

ANOVA **FDR**

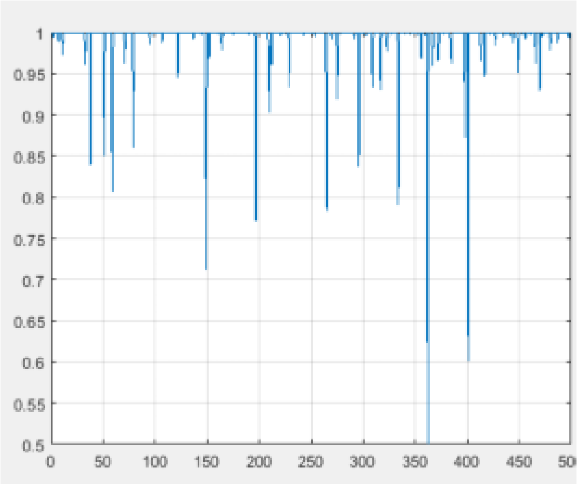

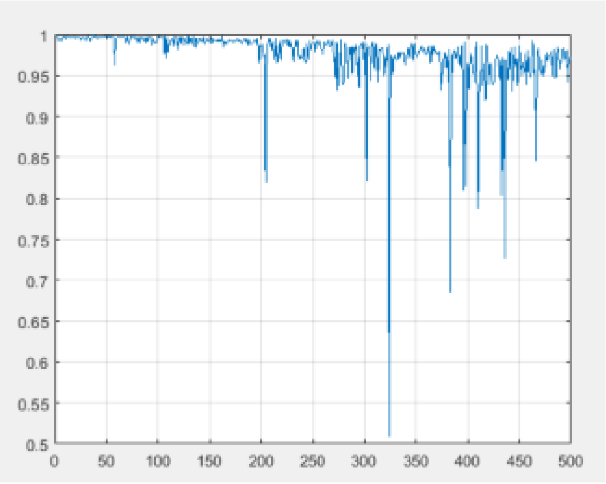

Ave **Gen-Score**

Figure 4 (continued) - GSE19491 dataset [69]

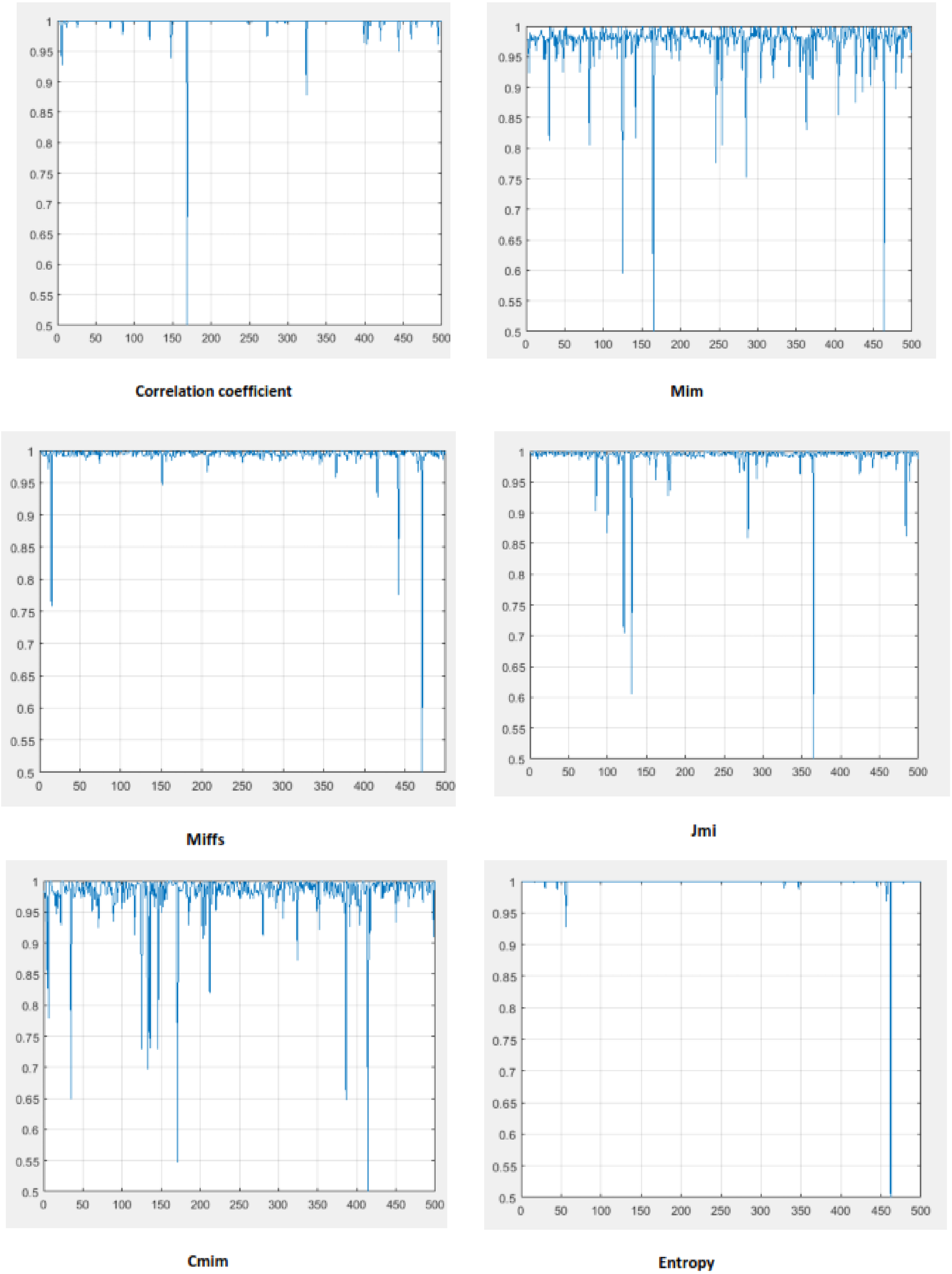

Figure 4 (continued) - GSE39939 dataset [72]

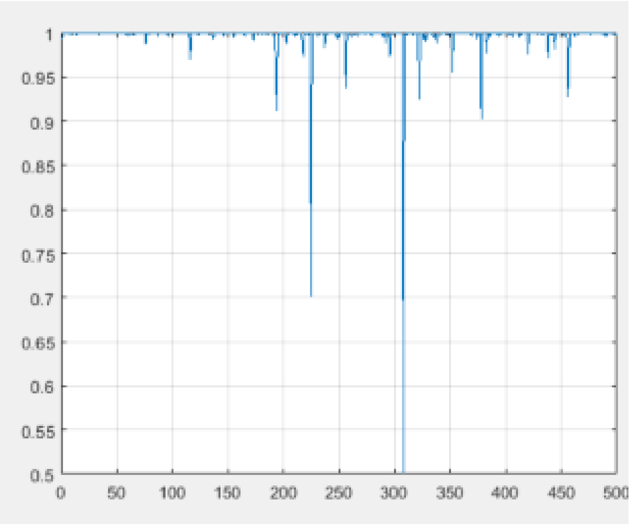

ANOVA

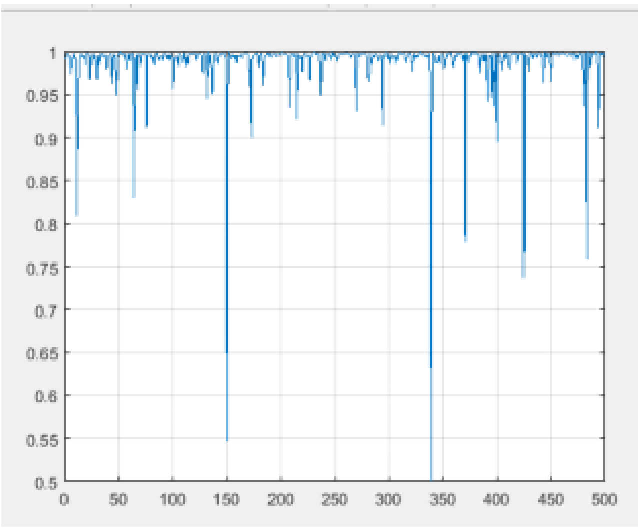

FDR

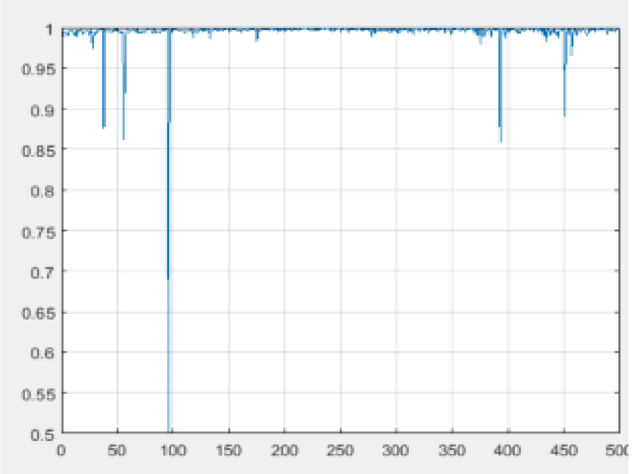

Ave

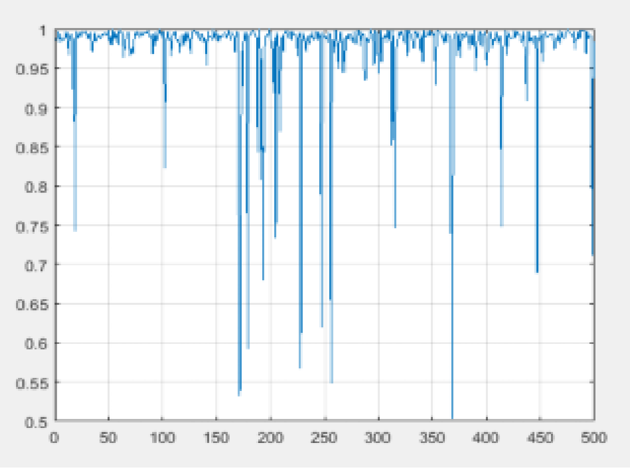

Gen-score

Figure 4 (continued) - GSE39939 dataset [72]

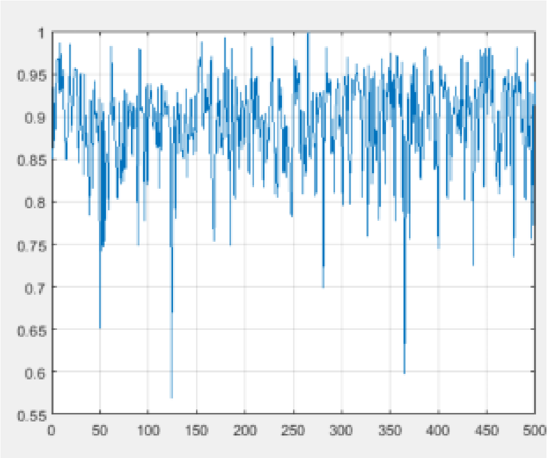

Correlation coefficient

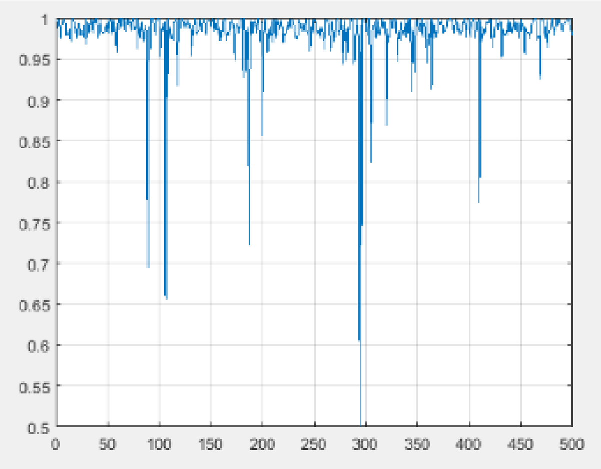

Mim

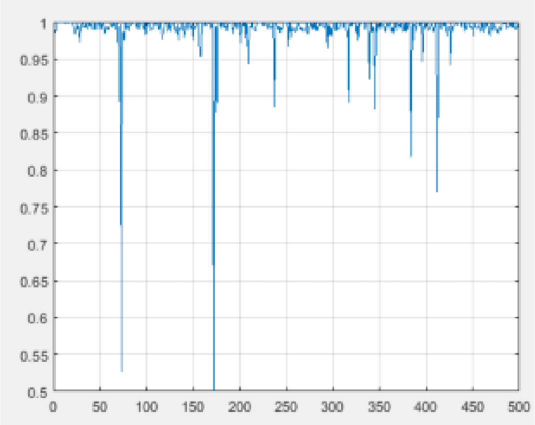

Miffs

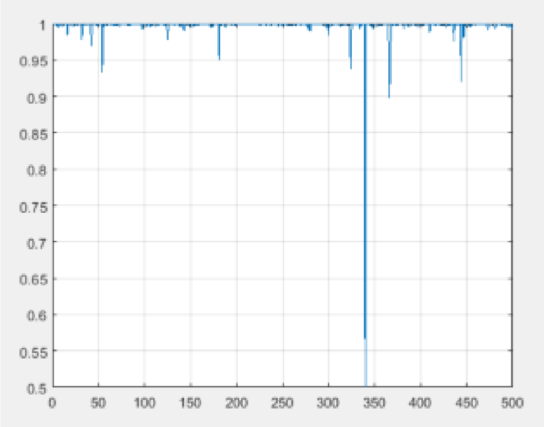

Jmi

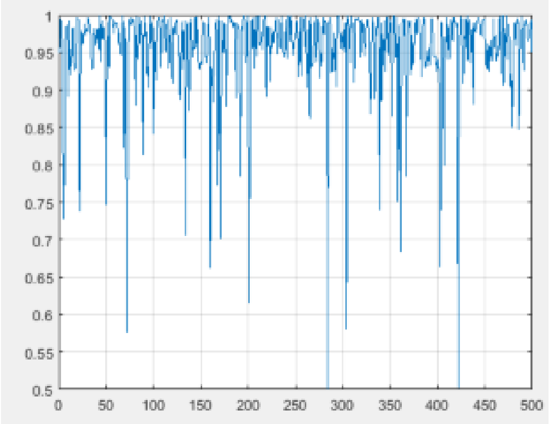

Cmim

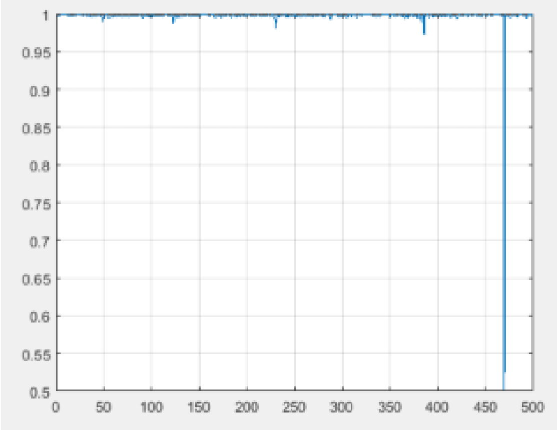

Entropy

Figure 4 (continued) - GSE19439 dataset [70]

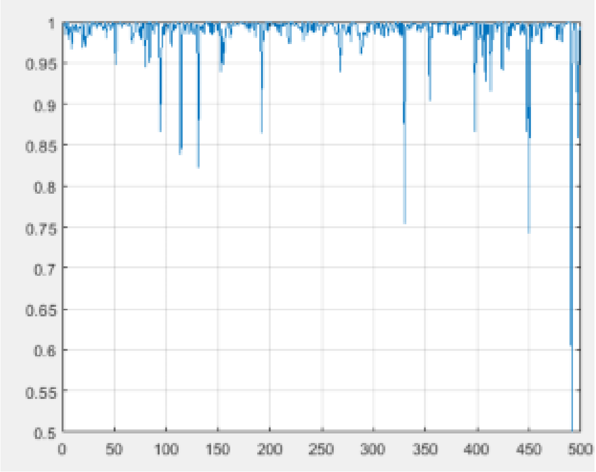

ANOVA

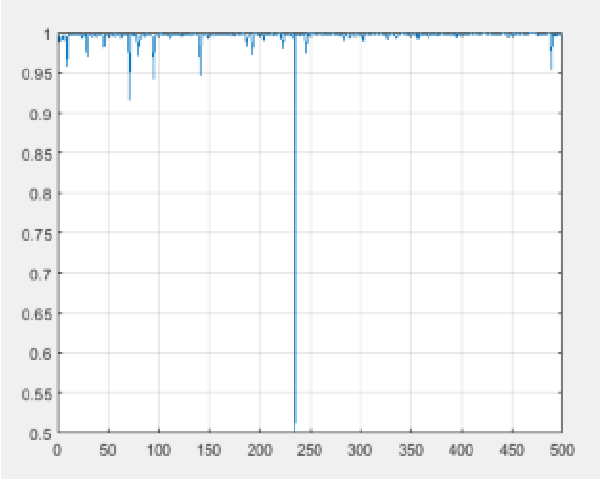

FDR

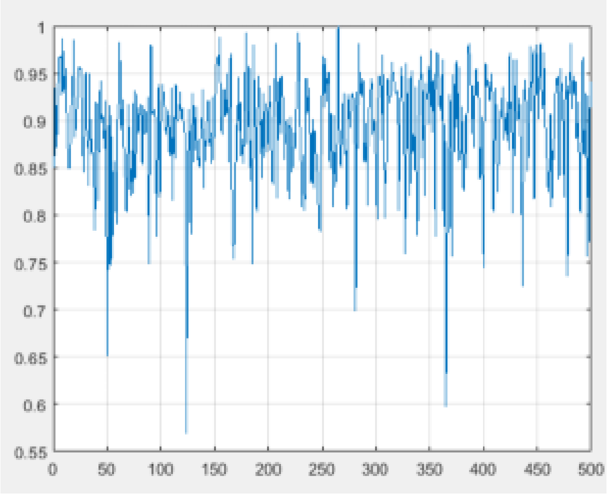

Ave

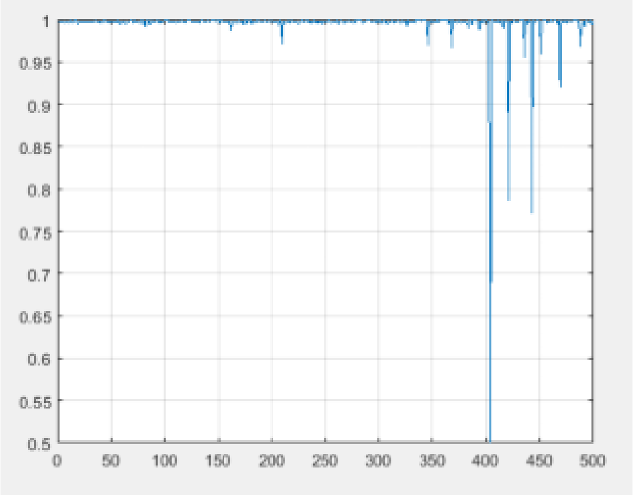

Gen-score

Figure 4 (continued) - GSE19439 dataset [70]

Using wrapping feature selection method, SFFS

In figures 3 and 5, the rows represent features (genes), and the columns indicate the importance level of each feature. The SFFS plot is used for feature selection in machine learning models, including the identification of top genes in microarray studies. It visually depicts how model performance changes with increasing numbers of selected features (e.g., genes). This helps researchers determine which additional genes enhance model performance, aiding in the selection of optimal feature combinations for biological analyses.

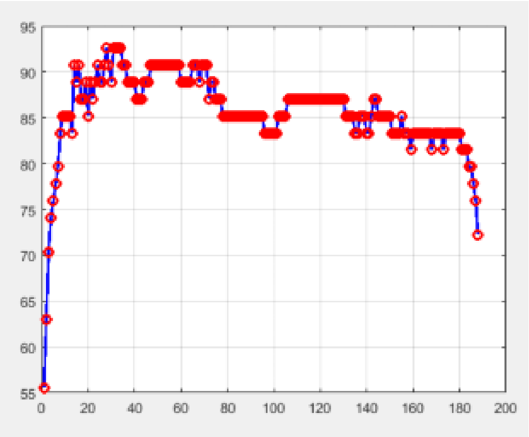

Correlation coefficient

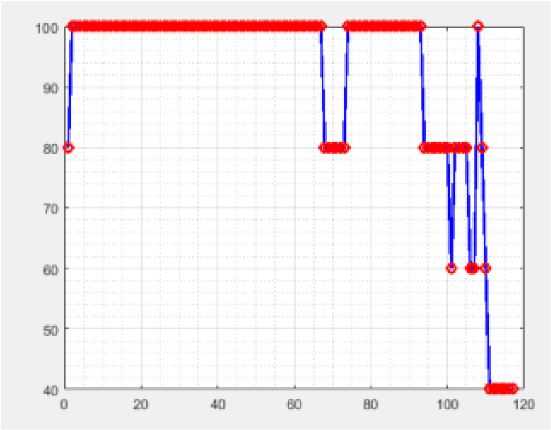

Mim

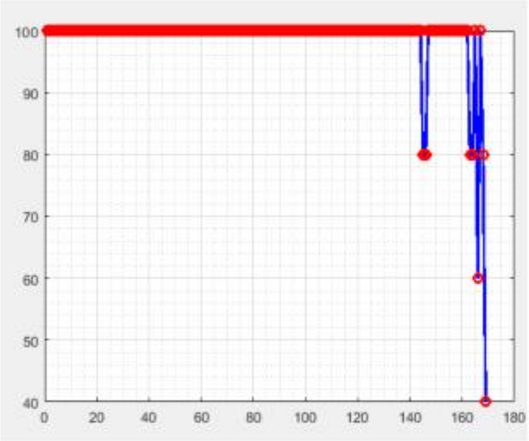

Miffs

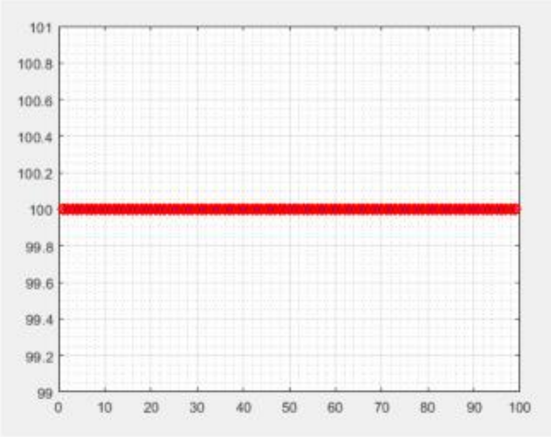

Jmi

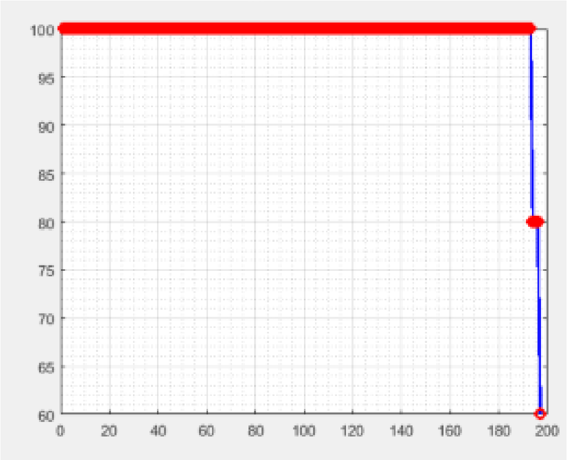

Cmim

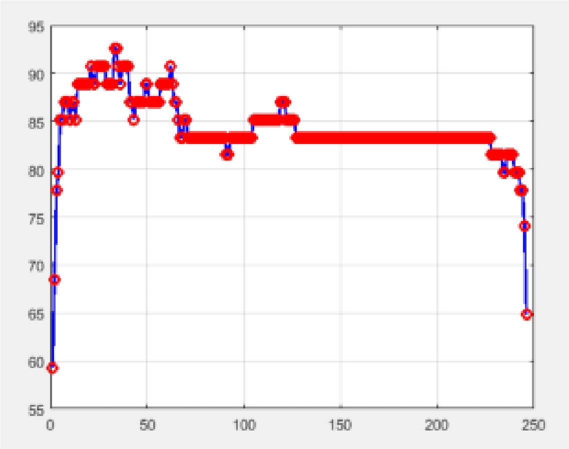

Entropy

Figure 6 Applying SFFS feature selection method on 500 genes - GSE37250 dataset [72]

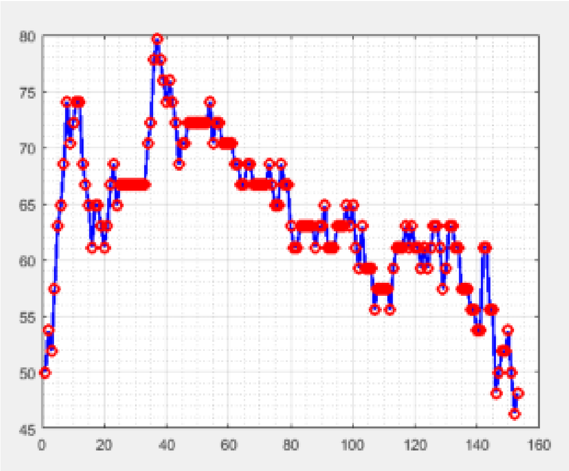

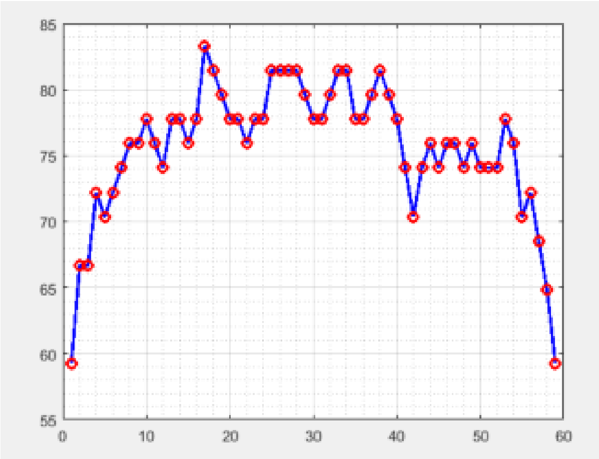

ANOVA **FDR**

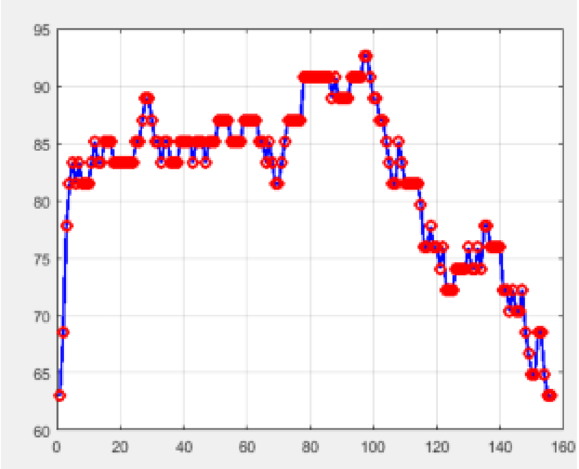

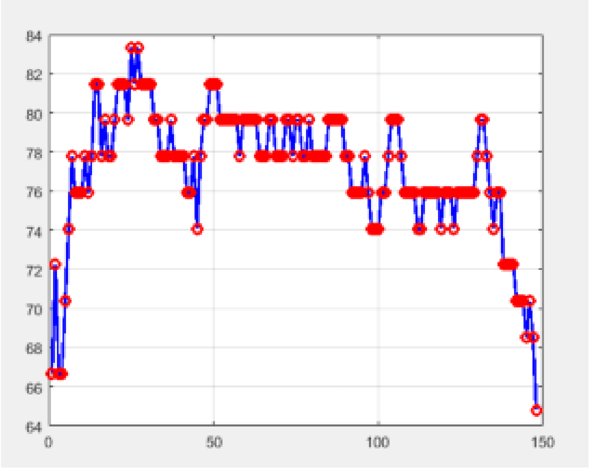

Ave **Gen-score**

Figure 6 (continued) - GSE37250 dataset [72]

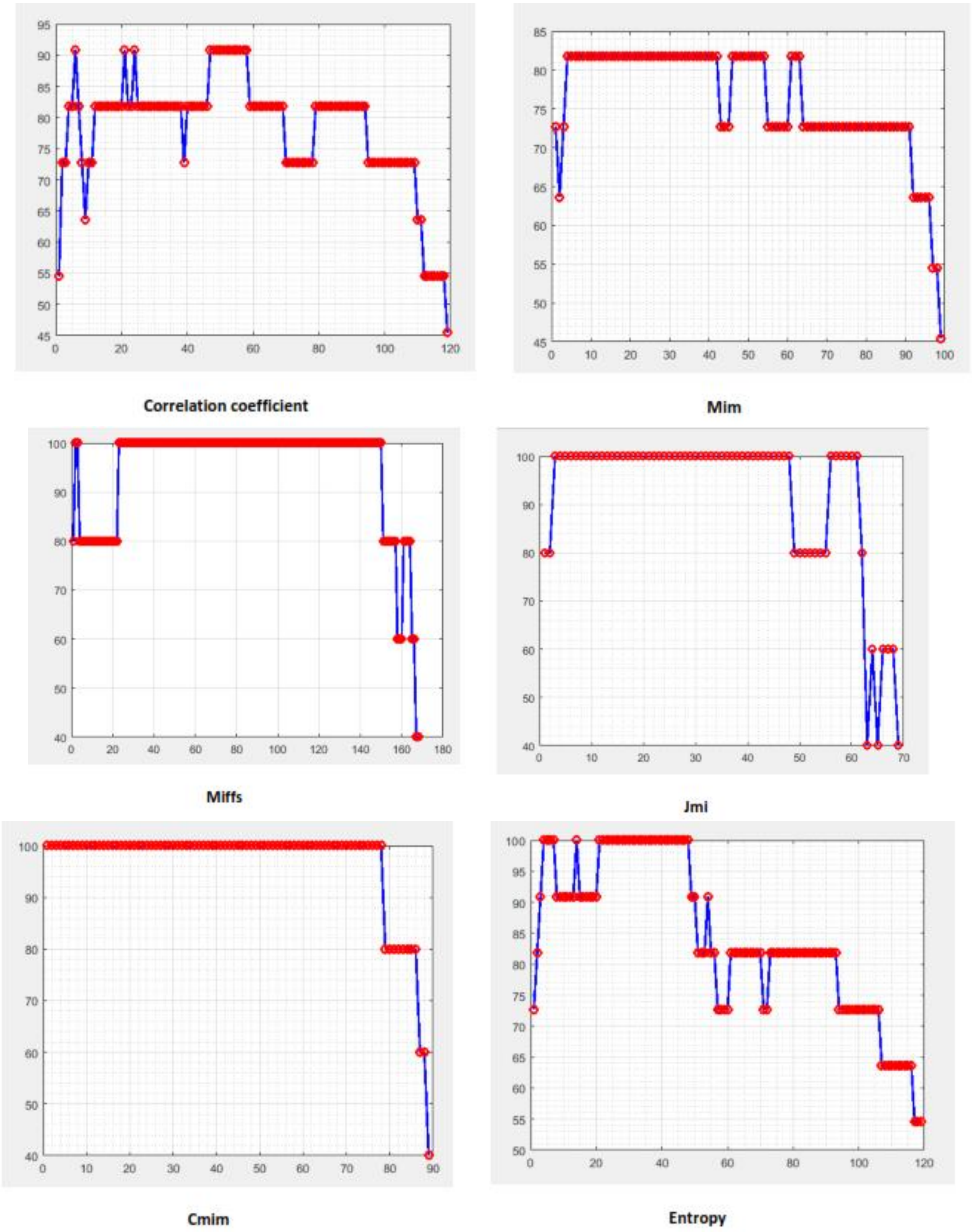

Figure 6 (continued) - GSE28623 dataset [70]

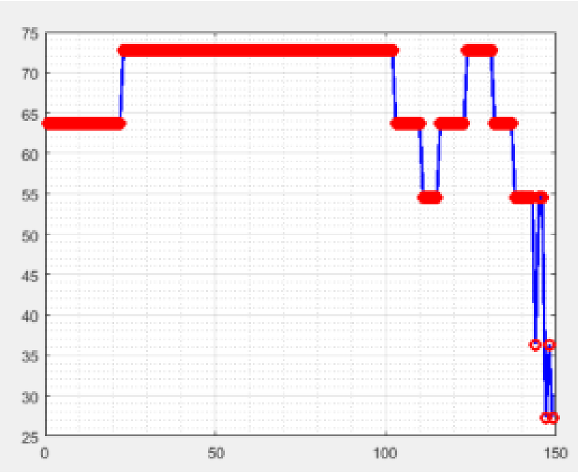

ANOVA

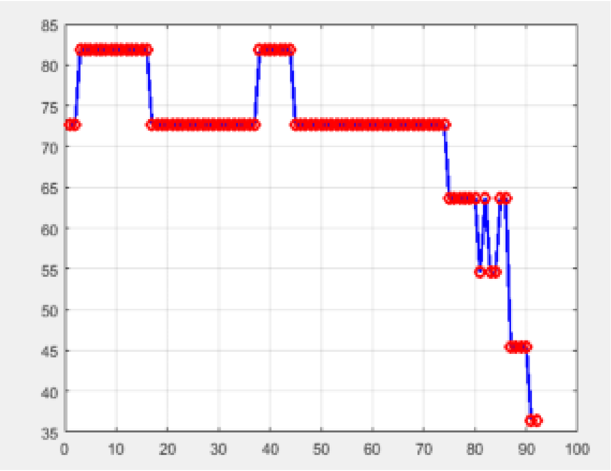

FDR

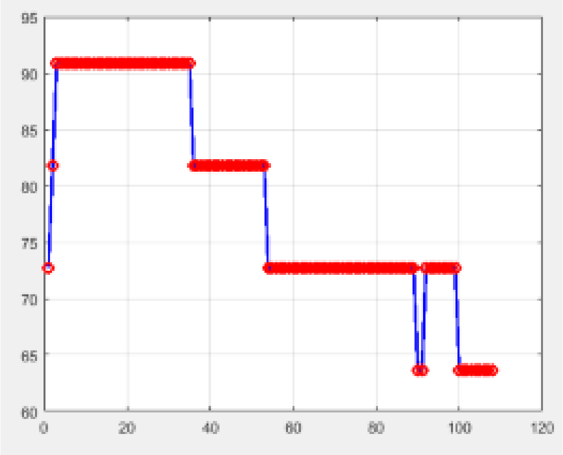

Ave

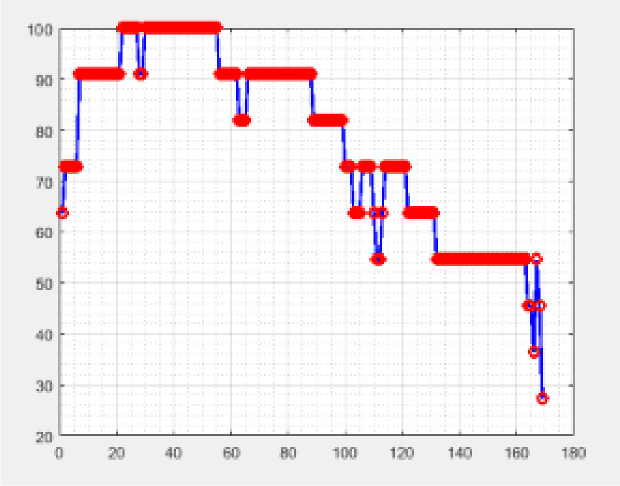

Gen-score

Figure 6 (continued) - GSE28623 dataset [70]

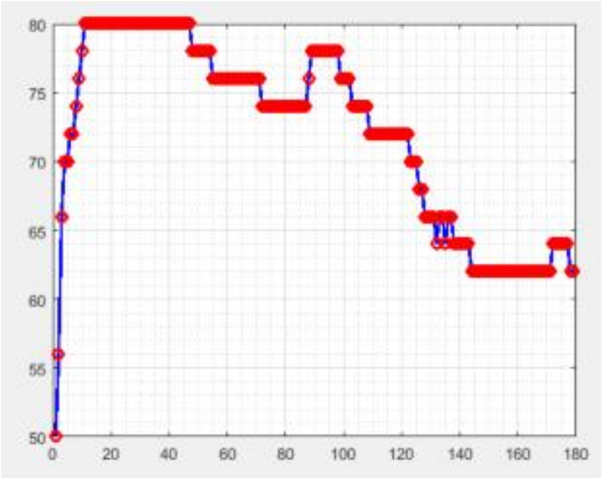

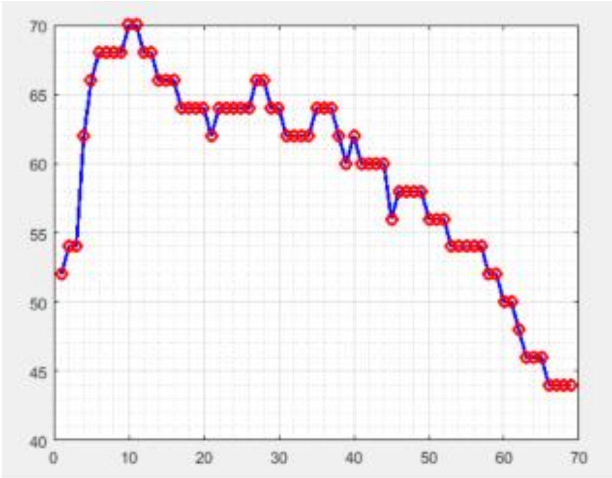

Correlation coefficient **Mim**

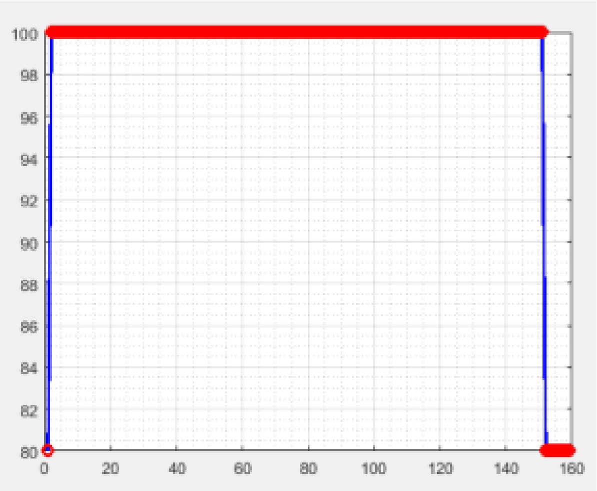

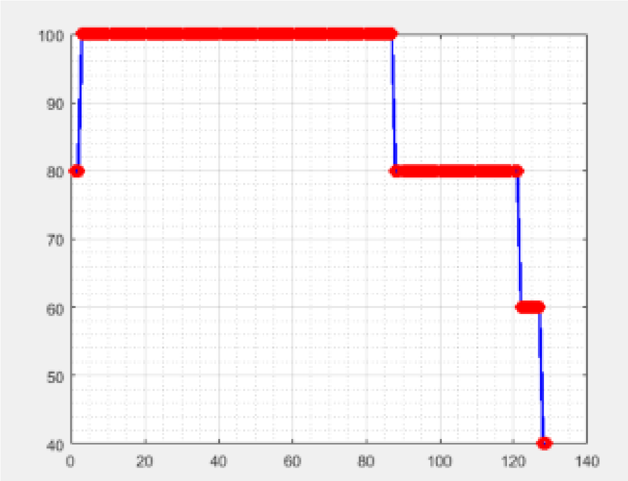

Miffs **Jmi**

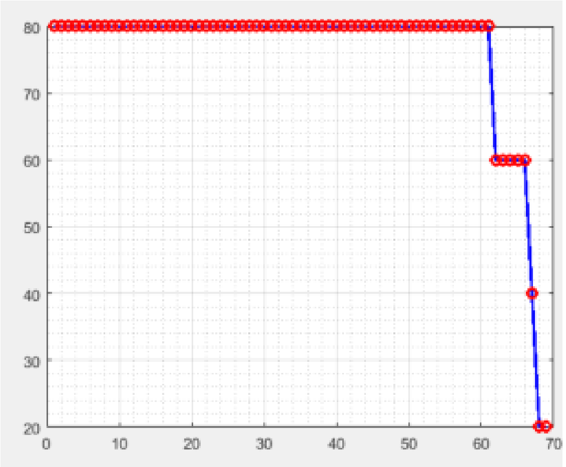

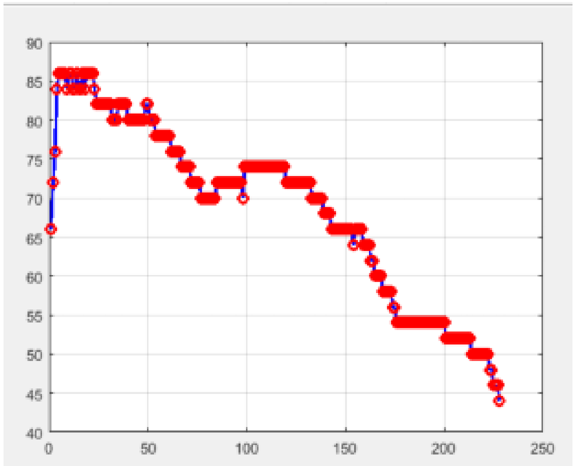

Cmim **Entropy**

Figure 6 (continued) - GSE19491 dataset [69]

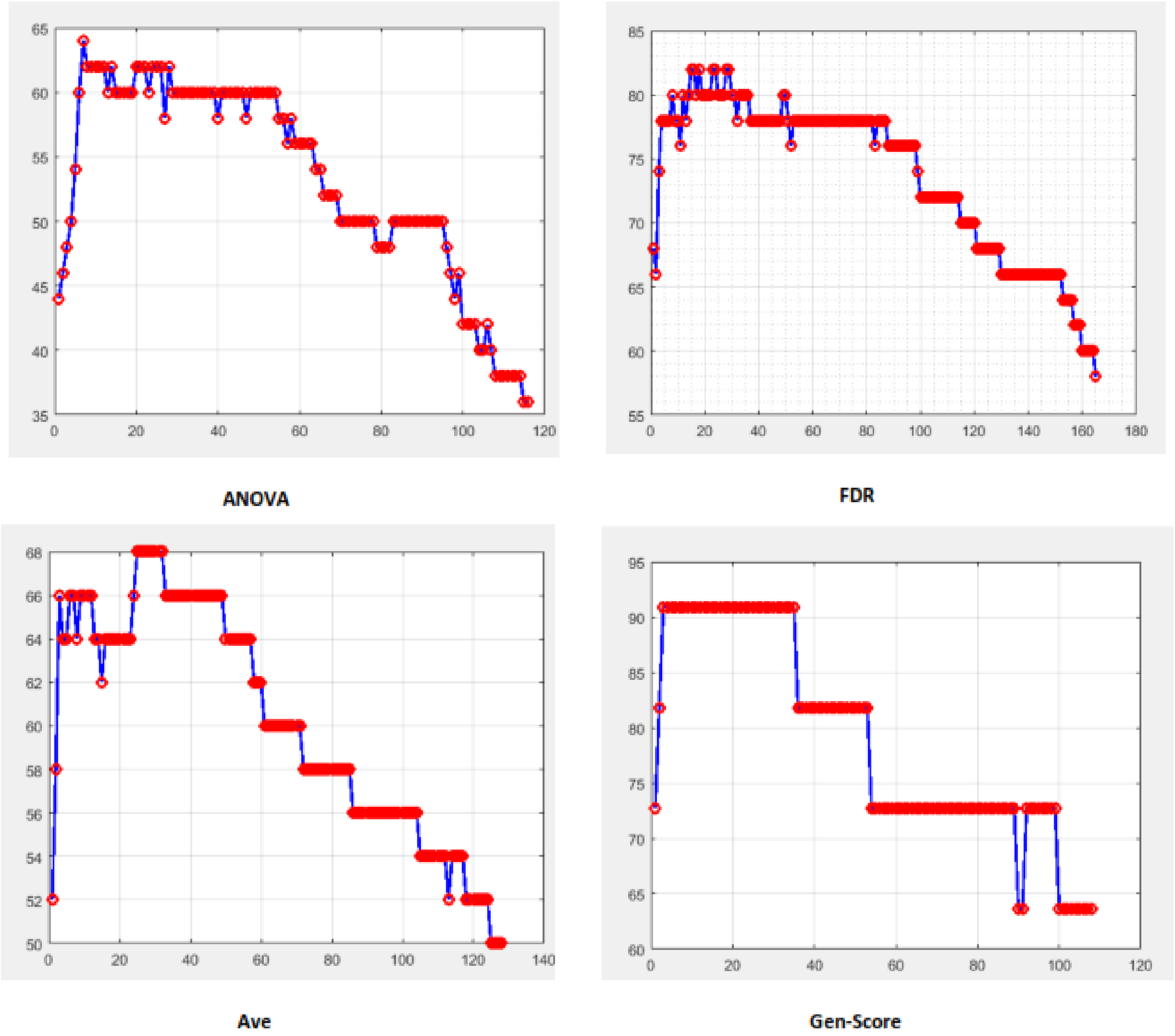

Figure 6 (continued) - GSE19491 dataset [69]

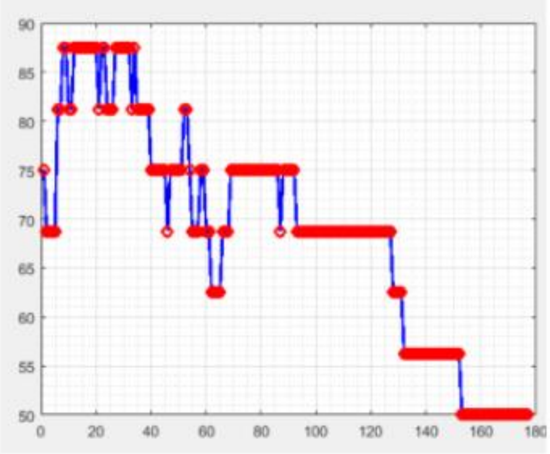

Correlation coefficient

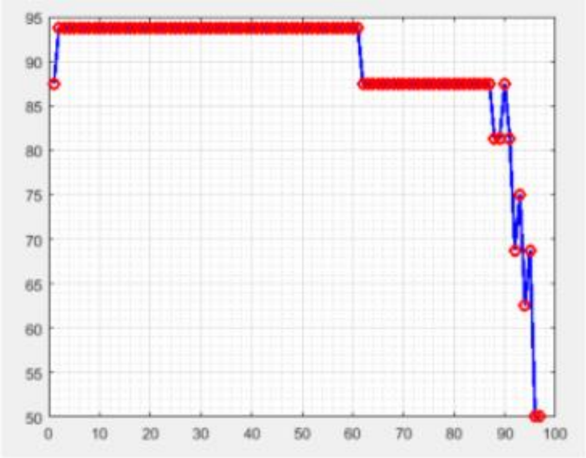

Mim

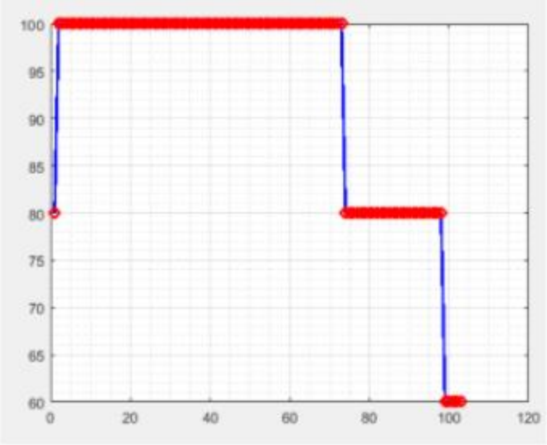

Miffs

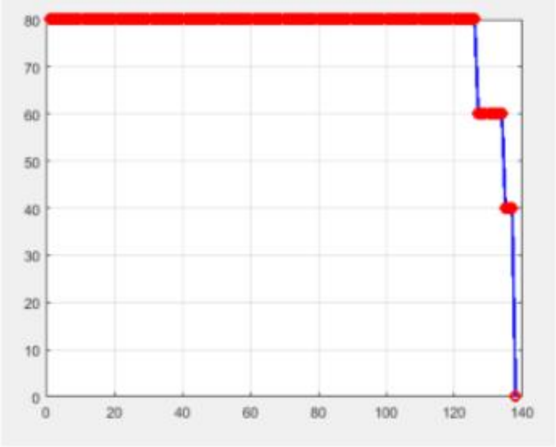

Jmi

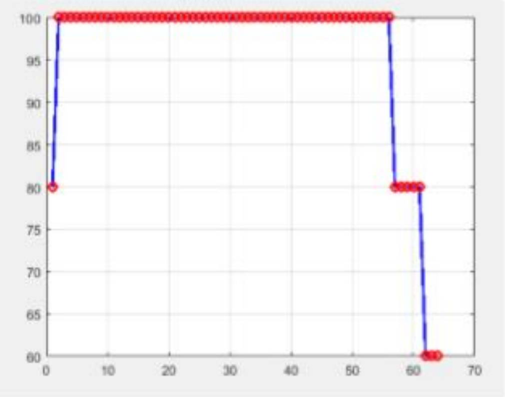

Cmim

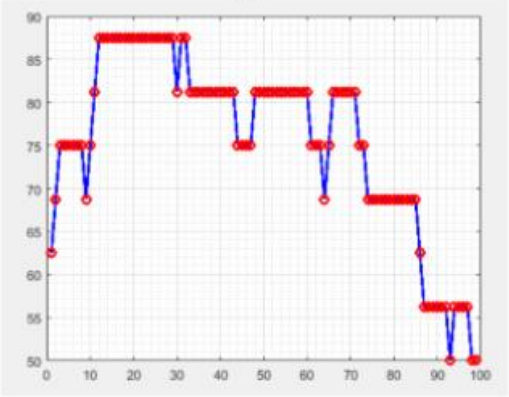

Entropy

Figure 6 (continued) - GSE39939 dataset [72]

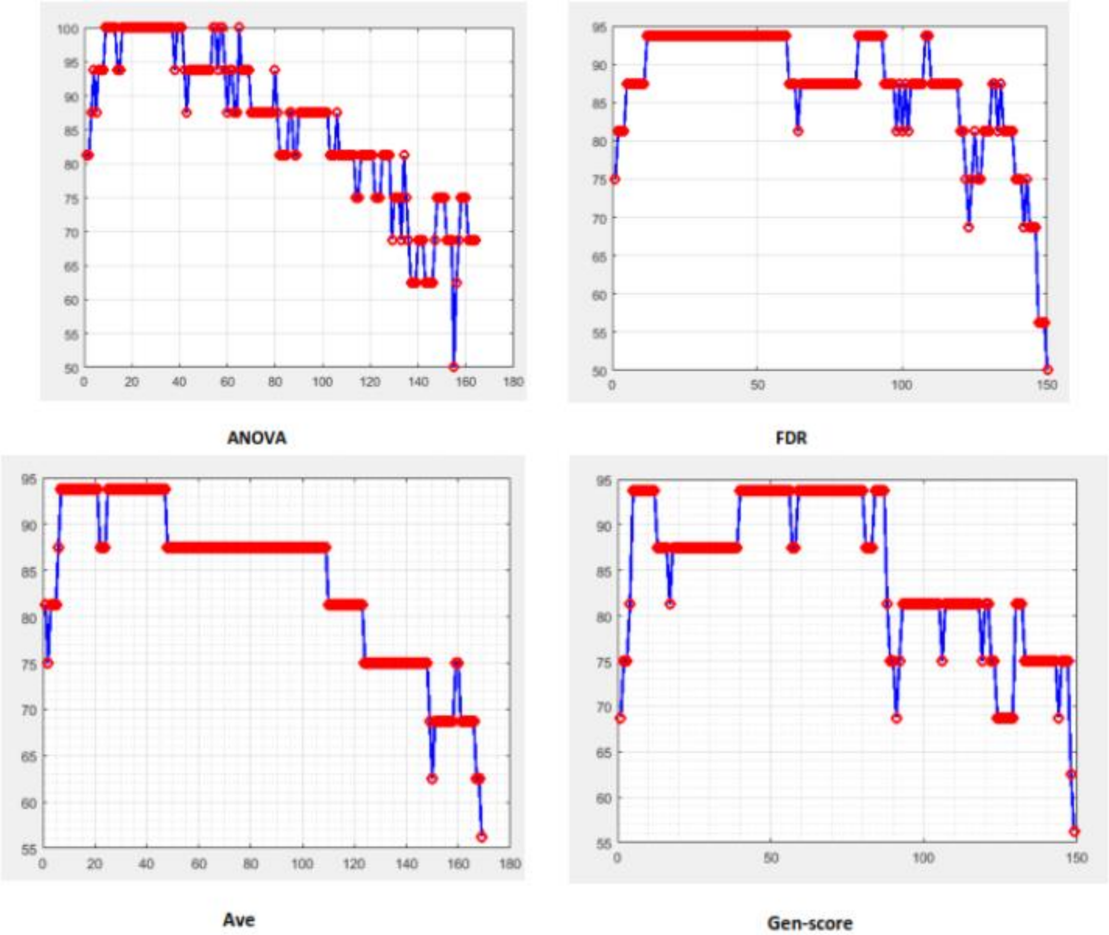

Figure 6 (continued) - GSE39939 dataset [72]

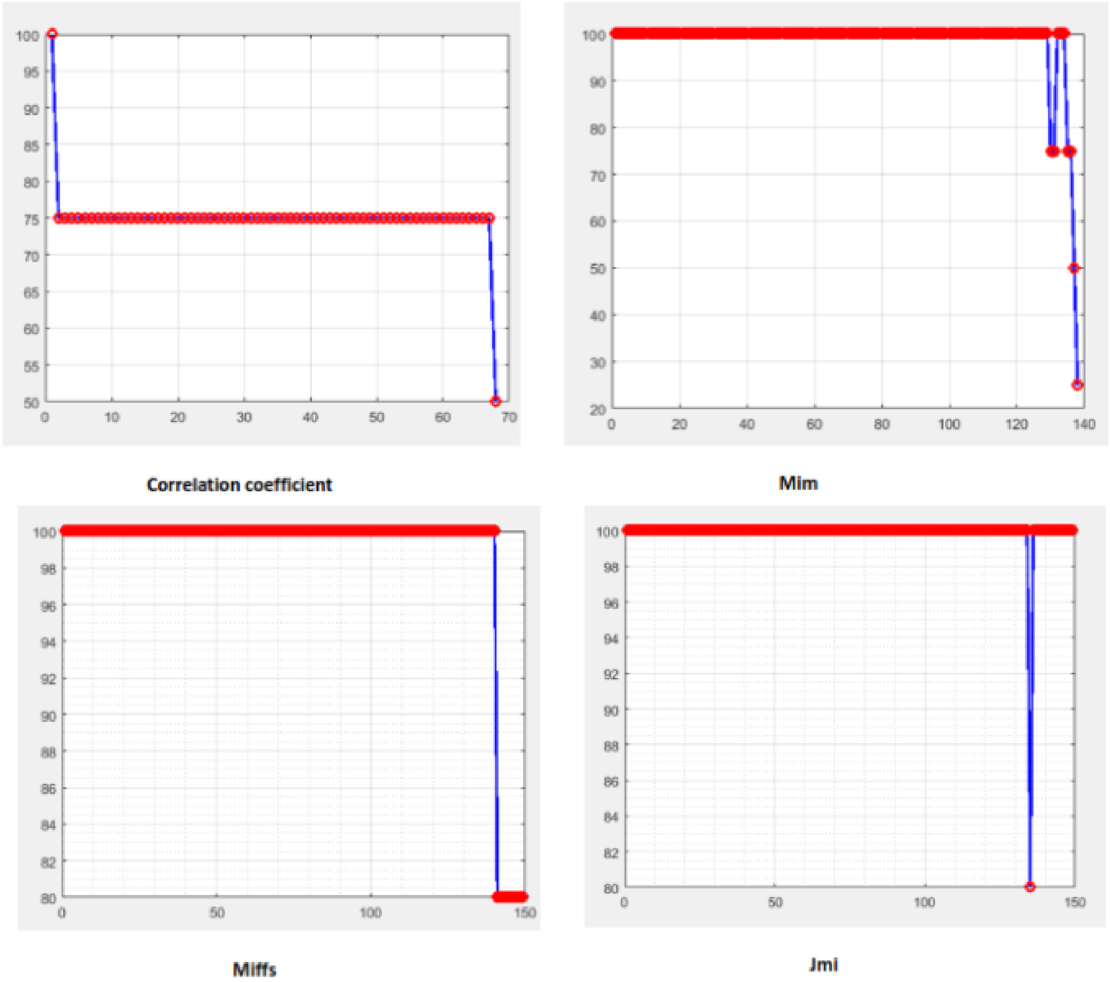

Figure 6 (continued) - GSE19439 dataset [70]

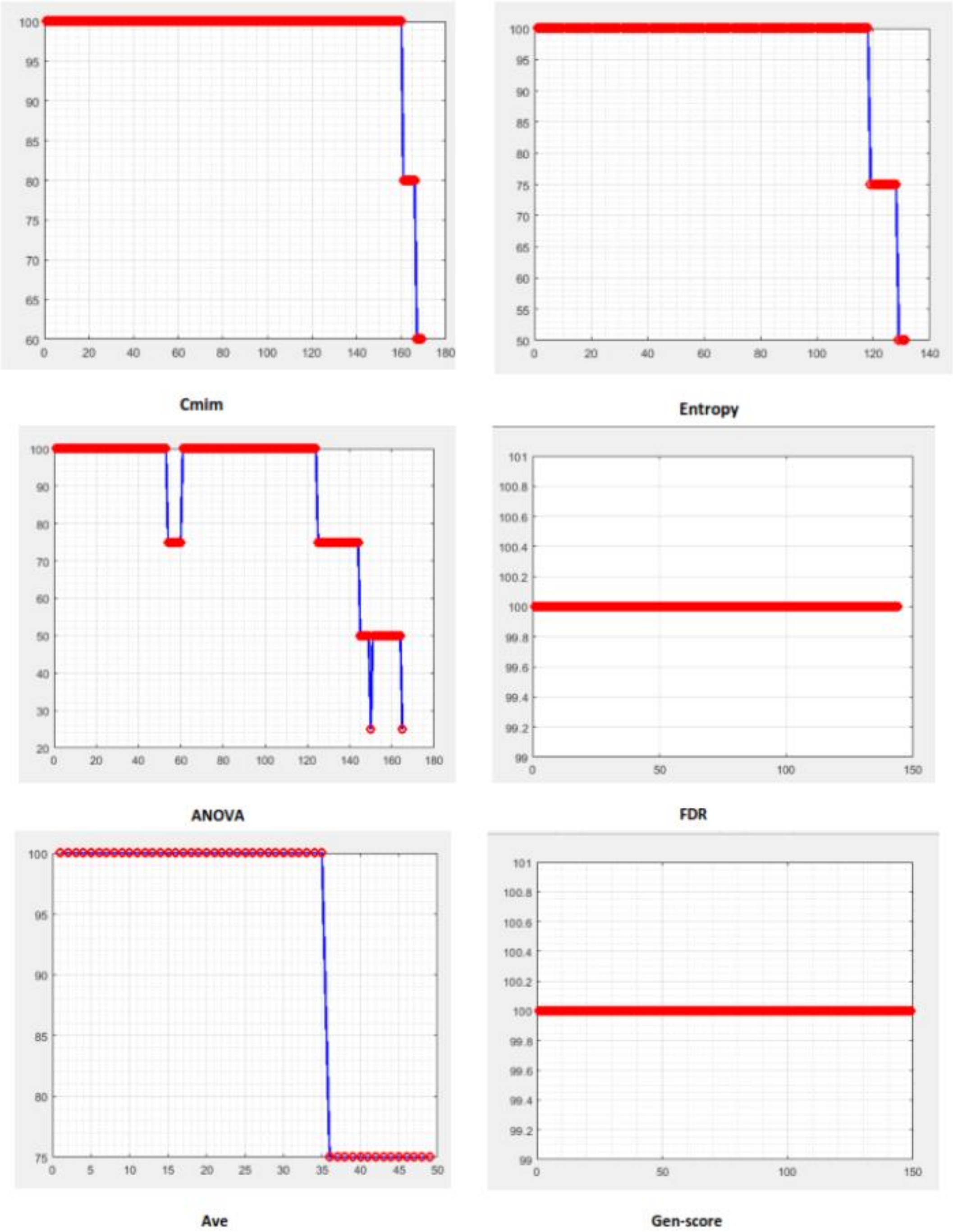

Figure 6 (continued) - GSE19439 dataset [70]

Appendix E. A tabular overview of gene interactions to create a communication table.
A tabular overview of gene interactions to create a communication table is shown in the links below.
https://github.com/somaieh68/appendix/blob/main/Appendix%20D%2C%20gene%20interactions2.pdf
